# A Unified Framework for Statistical Inference and Power Analysis of Single and Comparative $F_{\beta }$ Scores

**DOI:** 10.1002/sim.70557

**Published:** 2026-04-28

**Authors:** Chih‐Yuan Hsu, Qi Liu, Yu Shyr

**Affiliations:** ^1^ Department of Biostatistics Vanderbilt University Medical Center Nashville Tennessee USA; ^2^ Center for Quantitative Sciences Vanderbilt University Medical Center Nashville Tennessee USA

**Keywords:** $F_{1}$ score, $F_{\beta }$ score, hypothesis testing, interval estimation, power and sample size calculation

## Abstract

Machine learning and artificial intelligence are increasingly applied to medical diagnostics and clinical decision‐making. To evaluate model performance, the $F_{1}$ score and its generalized form, the $F_{\beta }$ score, are widely used as they balance precision and sensitivity. However, rigorous statistical inference and power analysis for the $F_{1}$ and $F_{\beta }$ scores remain limited. In this study, we propose psF1, a unified and comprehensive framework for interval estimation, hypothesis testing, and power and sample size calculation for both single and comparative $F_{1}$ and $F_{\beta }$ scores. psF1 leverages exact probability distributions as well as approximations for large sample sizes to provide valid statistical inference and power analyses. Extensive simulations demonstrate the accuracy and robustness of psF1 across a range of sensitivity, precision, and sample size scenarios. We further showcase its practical utility through real‐world biomedical classification tasks. This framework enables principled evaluation and comparison of classifiers using $F_{1}$ and $F_{\beta }$ scores with reliable uncertainty quantification and informed sample size planning. psF1 is freely available at http://github.com/cyhsuTN/psF1.

## 1 Introduction

In recent years, machine learning and artificial intelligence‐based systems have become increasingly important in supporting healthcare professionals with medical diagnostics and clinical decision‐making. They assist in predicting, detecting, and classifying a wide range of conditions, including heart disease [1, 2], skin and lung cancers [3, 4], COVID‐19 [5], and complications and readmissions after spine surgery [6]. As these tools become more integrated into clinical workflows, rigorous evaluation of their predictive and classification performance is essential to ensure reliability and clinical utility.

Accuracy, area under the curve (AUC), and the $F_{1}$ score are among the most commonly used metrics for evaluating classification performance. Accuracy measures the proportion of correct predictions among all classification decisions, while AUC quantifies performance by measuring the area under the receiver operative characteristic (ROC) curve. However, both accuracy and AUC can be misleading in highly imbalanced data [6]. For example, in the Noninvasive Examination of Trisomy study, where the majority of pregnancies were non‐trisomy [7], results are dominated by the negative cases. Standard screening therefore achieved both high accuracy and high AUC despite poor sensitivity, generating an overly optimistic perception of performance. In contrast, the $F_{1}$ score, defined as the harmonic mean of precision (also known as positive predictive value, PPV) and sensitivity (also known as recall), offers a more balanced evaluation in imbalanced data. Compared to accuracy and AUC, the $F_{1}$ score is directly reduced by low sensitivity or low precision, providing a more realistic assessment of performance and stronger discriminatory power when comparing different classifiers. The generalized $F_{\beta }$ score introduces a user‐defined parameter β to adjust the relative importance of sensitivity (β > 1) versus precision (β < 1), providing flexibility depending on the application. Since both the $F_{1}$ and $F_{\beta }$ scores range from 0 to 1, with higher values indicating better classification performance, most studies have ranked classifier performance solely based on these scores, often without performing formal statistical inference or hypothesis testing [8]. This lack of rigorous evaluation undermines the reliability of comparisons across classifiers and may lead to overconfident or unsupported conclusions.

Several methods have been proposed for statistical inference of the $F_{1}$ score, adopting either a frequentist or a Bayesian approach, with a focus on interval estimation [9, 10, 11, 12]. Conditioned on the sum of true positives, false positives, and false negatives (Table 1), Flores et al. [9] derived the asymptotic normal distribution of the $F_{1}$ score using a simple transformation of the $F_{1}$ score [13]. Based on their derivations, Lam et al. [10] constructed confidence intervals (CIs) using the Clopper–Pearson method and the Wald method, and further proposed two new CIs using the Wilson direct method and Wilson indirect method by modifying the variance of the asymptotic normal distribution. On the Bayesian side, Goutte and Gaussier [11] assumed that precision and sensitivity arose from independent gamma random variables and expressed the $F_{1}$ score as the combination of these gamma random variables. Using this framework, Wang et al. [12] derived the posterior density function of the $F_{1}$ score and constructed corresponding credible intervals (CrIs). Furthermore, these Bayesian methods were further extended to develop CrIs for comparing the $F_{1}$ scores of two independent classifiers [11, 14].

**TABLE 1:** Confusion matrix.

		Predicted		
		Negative	Positive	
Actual	Negative	a (TN)	b (FP)	N−S
	Positive	c (FN)	d (TP)	S
		a+c	b+d	N

Abbreviations: TN: true negatives; FN: false negatives; FP: false positives; TP: true positives; N: the total number of classification instances; S: the number of positives.

Although CIs and CrIs can be adapted for hypothesis testing, few formal hypothesis testing methods have been developed for evaluating the $F_{1}$ score or comparing $F_{1}$ scores between two classifiers. To our knowledge, no dedicated testing methods have been proposed specifically for assessing a single $F_{1}$ score. For comparing $F_{1}$ scores between two classifiers, a potential method is the permutation‐based approach, inspired by Yeh [15], which constructs an empirical null distribution of the $F_{1}$ score difference. However, the accuracy of *p* values depends on the number of permutations and can be time‐consuming when the sample size is large. More recently, Takahashi et al. [16] proposed formal test statistics for comparing $F_{1}$ scores in a paired study design, based on the large sample multivariate central limit theorem. Their method can also be reduced to the case of two independent classifiers by setting the covariance term in the test statistics to zero [16].

Compared to interval estimation and hypothesis testing, there is currently a lack of methods for conducting power and sample size calculations specifically for the $F_{1}$ score. A straightforward and intuitive approach is to use simulation‐based procedures, which repeatedly generate data and apply existing testing methods to estimate empirical power. However, these simulation‐based procedures are often computationally intensive and time‐consuming, limiting their practicality for routine study design.

In summary, statistical methods for the $F_{1}$ score remain limited, especially in hypothesis testing and power and sample size calculation, and there are no available methods for the more general $F_{\beta }$ score. In this study, we propose a unified and comprehensive statistical framework, psF1, for evaluating the $F_{1}$ score of a single classifier and for comparing $F_{1}$ scores between two classifiers. psF1 supports interval estimation, hypothesis testing, and power and sample size calculations. Furthermore, we extend psF1 to the $F_{\beta }$ score, the first approach for this generalization, allowing flexible weighting of precision and sensitivity. psF1 leverages exact probability distributions and normal approximations for large sample sizes, offering both robustness and computational efficiency.

This remainder of this paper is organized as follows. Section 2.1 introduces the new methods for confidence interval, hypothesis testing, and power and sample size calculation for assessing the $F_{1}$ score of a single classifier. Section 2.2 presents analogous methods for comparing $F_{1}$ scores between two classifiers. Section 2.3 describes the normal approximation of $F_{1}$ scores. Section 2.4 extends these methods to the $F_{\beta }$ score. Section 3 reports the results of simulation studies. Section 4 presents the real applications of the proposed methods. Finally, Section 5 summarizes the contributions of this study and suggests directions for future research.

## 2 Methods

The $F_{1}$ score is defined as the harmonic mean of precision $p_{p}$ and sensitivity $\;p_{s}$, given by $\;F_{1}=F_{1}\left(p_{s},p_{p}\right)=2p_{s}p_{p}/\left(p_{s}+p_{p}\right)$. In the context of a confusion matrix where S is the number of actual positives, N−S the number of actual negatives, d the number of true positives, and b the number of false positives (Table 1). The $F_{1}$ score can be estimated using the formula $f_{1}=\frac{2d}{b+d+S}$ [9, 10]. Despite the widespread use of the $F_{1}$ score, its statistical properties are less frequently addressed. This leads to several open statistical questions, including how to obtain reliable interval estimates for the $F_{1}$ score, how to formally compare $F_{1}$ scores between classifiers through hypothesis testing, and how to perform power and sample size calculations when the $F_{1}$ score is the target metric.

To address these issues, we propose psF1, a unified framework that supports interval estimation, hypothesis testing, and power and sample size calculation for the $F_{1}$ and $F_{\beta }$ scores. All methods included in psF1 are implemented in the R package on GitHub: http://github.com/cyhsuTN/psF1. Artificial examples for statistical inference and study design and detailed usage instructions are provided in the Supporting Information. A summary of these methods and their corresponding R functions is listed in Table 2, with detailed descriptions provided below.

**TABLE 2:** A summary of methods and corresponding R functions in psF1 supporting interval estimation, hypothesis testing, and study design for single classifiers and comparisons between two classifiers.

	Single classifier	Two classifiers' comparison
Interval estimation	Confidence interval for $F_{\beta }$ scores R function: *ciFbOne*	Confidence interval for comparative $F_{\beta }$ scores R function: *ciFbTwo*
Hypothesis testing	Given $p_{s0}$ and $p_{p0}$, $H_{0}:F_{1}=F_{1}\left(p_{s0},p_{p0}\right)$ vs. $H_{1}:F_{1}\neq F_{1}\left(p_{s0},p_{p0}\right)$ Without specifying $p_{s0}$ and $p_{p0}$, $H_{0}:F_{1}=F_{1,0}$ vs. $H_{1}:F_{1}\neq F_{1,0}$ (conservative) R function: *testFbOne*	$H_{0}:F_{1,1}=F_{1,2}$ vs. $H_{1}:F_{1,1}\neq F_{1,2}$ $F_{1,1}=F_{1,2}={\hat{F}}_{1,0}$, where ${\hat{F}}_{1,0}$ = $F_{1}\left({\bar{p}}_{s},{\bar{p}}_{p}\right)$ $F_{1,1}=F_{1,2}=F_{1,0}$for all $F_{1,0}$ without specifying underlying sensitivity and precision values (conservative) R function: *testFbTwo*
Study design	Power and sample size calculation Correspond to the hypothesis tests above R function: *powerFbOne*	Power and sample size calculation Correspond to the hypothesis tests above R function: *powerFbTwo*

### 2.1 Methods for the $F_{1}$ Score of a Single Classifier

#### 2.1.1 Confidence Interval

To construct a confidence interval for the $F_{1}$ score of a classifier, we first need to derive the distribution of $f_{1}$, based on a given precision $p_{p}$ and sensitivity $p_{s}$. We assume that the classifier's decisions for individual instances are independent, implying that b and d are independent. We model b and d using binomial distributions: b ˜ $\mathrm{Bin}\;\left(N-S,p_{\alpha }\right)$ and d ˜ $\mathrm{Bin}\left(S,p_{s}\right)$. The $p_{\alpha }$ satisfies $E\left(f_{1}\right|\;S,N-S,p_{s},p_{\alpha })=F_{1}$, where $F_{1}=F_{1}\left(p_{s},p_{p}\right)$. Specifically, the $p_{\alpha }$ is the solution to Equation (1):

(1) $$\sum_{d=0}^{S}\sum_{b=0}^{N-S}\frac{2d}{b+d+S}\mathrm{Bin}\left(b;N-S,p_{\alpha }\right)\mathrm{Bin}\left(d;S,p_{s}\right)=F_{1}.$$

When $p_{s}$ and $p_{p}$ are specified, the $F_{1}$ score is determined. Achieving this $F_{1}$ score on average requires controlling the distributions of both true positives d and false positives b. The $p_{s}$ governs the distribution of true positives given S, while the $p_{\alpha }$ controls the distribution of false positives given N−S. Consequently, the value of $p_{\alpha }$ depends not only on $p_{s}$ and $p_{p}$ but also on S and N. When S is fixed, the value of $p_{\alpha }$ decreases as N−S increases. For example, when $p_{p}$ = 0.6, $p_{s}$ = 0.9, the value of $p_{\alpha }$ are 0.40, 0.15, and 0.09 for N−S = 30, 80, and 130, respectively, with S = 20. When S and N are large, $p_{\alpha }\approx \frac{S}{N-S}\left\{p_{s}\left(2F_{1}^{-1}-1\right)-1\right\}$ (See Section 2.3 for details). With $p_{\alpha }$ computed and $p_{s}$ specified, all values of $f_{1}=2d/\left(b+d+S\right)$ and corresponding probabilities can be obtained by the two binomial distributions. The probability mass function of $f_{1}=f$ can be explicitly expressed as follows:

(2) $$g\left(f;p_{s},p_{p}\right)=\sum_{\left(b,d\right)\in Q\left(f\right)}\mathrm{Bin}\left(b;N-S,p_{\alpha }\right)\mathrm{Bin}\left(d;S,p_{s}\right),$$

where $Q\left(f\right)=\left\{\left(b,d\right):\frac{2d}{b+d+S}=f,d\in {\mathbb{Z}}_{S},b\in {\mathbb{Z}}_{N-S}\right\}$ and $f\in {ℱ}_{N,S}=\left\{\frac{2d}{b+d+S}:d\in {\mathbb{Z}}_{S},b\in {\mathbb{Z}}_{N-S}\right\}$. ${\mathbb{Z}}_{n}=\left\{0,1,\ldots ,n\right\}$. Therefore, we can construct a 100($1-\alpha^{*}$)% confidence interval for the $F_{1}$ score of the classifier given observed values $b=b_{\mathrm{obs}}$ and $d=d_{\mathrm{obs}}$, where $1-\alpha^{*}$ is a pre‐specified confidence level. This interval is denoted as [L,U], where

(3) $$\begin{array}{cc} & L=\max\left\{l\in {ℱ}_{N,S}:\sum_{f\leq l}g\left(f;{\tilde{p}}_{s},{\tilde{p}}_{p}\right)\leq \alpha^{*}/2\right\}\;\;\text{and}\\ & \;\;U=\min\left\{u\in {ℱ}_{N,S}:\sum_{u\leq f}g\left(f;{\tilde{p}}_{s},{\tilde{p}}_{p}\right)\leq \alpha^{*}/2\right\}. \end{array}$$

Typically, we consider ${\hat{p}}_{s}=d_{\mathrm{obs}}/S$ and ${\hat{p}}_{p}=d_{\mathrm{obs}}/\left(b_{\mathrm{obs}}+d_{\mathrm{obs}}\right)$ as the estimates for $p_{s}$ and $p_{p}$. However, when N and S are small, the interval estimate may exhibit insufficient coverage probability especially when $b_{\mathrm{obs}}$ = 0. To address this issue, we set $b_{\mathrm{obs}}$ = 0.5 if $b_{\mathrm{obs}}$ = 0, and consider ${\tilde{p}}_{s}=\left(d_{\mathrm{obs}}+0.5\right)/\left(S+1\right)$ and ${\tilde{p}}_{p}=\left(d_{\mathrm{obs}}+0.5\right)/\left(b_{\mathrm{obs}}+d_{\mathrm{obs}}+1\right)$, which equal the posterior means of $p_{s}$ and $p_{p}$ under Jeffrey's non‐informative prior [11].

#### 2.1.2 Hypothesis Testing

Two types of hypothesis tests are considered for evaluating a classifier's $F_{1}$ score. One test evaluates whether the classifier's $F_{1}$ score differs from a target value defined by a specified sensitivity $p_{s0}$ and precision $p_{p0}$, with hypotheses $H_{0}:F_{1}=F_{1}\left(p_{s0},p_{p0}\right)$ vs. $H_{1}:F_{1}\neq F_{1}\left(p_{s0},p_{p0}\right)$. The other test examines whether the classifier's $F_{1}$ score differs from a given benchmark value $F_{1,0}$, which is not necessarily tied to any specific sensitivity and precision values. In this case, the hypotheses are $H_{0}:F_{1}=F_{1,0}$ vs. $H_{1}:F_{1}\neq F_{1,0}$.

##### 2.1.2.1 $H_{0}:F_{1}=F_{1}\left(p_{s0},p_{p0}\right)$ vs. $H_{1}:F_{1}\neq F_{1}\left(p_{s0},p_{p0}\right)$

First, we construct the null distribution of $f_{1}$ under the null hypothesis. Following the procedure in Section 2.1.1, the probability mass function of $f_{1}=f$ can be expressed as $g\left(f;p_{s0},p_{p0}\right)$. When we observe $f_{1}=f_{\mathrm{obs}}=2d_{\mathrm{obs}}/\left(b_{\mathrm{obs}}+d_{\mathrm{obs}}+S\right)$, the *p* value can be calculated by

(4) $$p_{v}\left(f_{\mathrm{obs}};p_{s0},p_{p0}\right)=\sum_{f\in R}g\left(f;p_{s0},p_{p0}\right),$$

where $R=\left\{f:C\left(f\right)\leq C\left(f_{\mathrm{obs}}\right)\;\right\}$ and $C\left(u\right)=\min\{\sum_{f\leq u}g\left(f;p_{s0},p_{p0}\right),\sum_{f\geq u}g\left(f;p_{s0},p_{p0}\right)\}$. R is the region of that f is more extreme than $f_{\mathrm{obs}}$. If $p_{v}\left(f_{\mathrm{obs}};p_{s},p_{p}\right)<\alpha^{*}$, we reject $H_{0}:F_{1}=F_{1,0}\left(p_{s},p_{p}\right)$, where $\alpha^{*}$ is a pre‐specified significance level. The *p* values for one‐sided hypothesis tests are presented in the Supporting Information.

##### 2.1.2.2 $H_{0}:F_{1}=F_{1,0}$ vs. $H_{1}:F_{1}\neq F_{1,0}$

Multiple combinations of $p_{s}$ and $p_{p}$ can yield the same $F_{1,0}$. Therefore, the *p* value for $H_{0}:F_{1}=F_{1,0}$ vs. $H_{1}:F_{1}\neq F_{1,0}$ is calculated by

(5) $$p_{v}\left(f_{\mathrm{obs}};F_{1,0}\right)=\underset{\left(p_{s},p_{p}\right)\in \Theta \left(F_{1,0}\right)}{\sup}p_{v}\left(f_{\mathrm{obs}};p_{s},p_{p}\right),$$

where $\Theta \left(F_{1,0}\right)=\left\{\left(p_{s},p_{p}\right):2p_{s}p_{p}/\left(p_{s}+p_{p}\right)=F_{1,0}\right\}$. Since $0\leq p_{s},p_{p}\leq 1$, we further express $\Theta \left(F_{1,0}\right)=\{\left(p_{s},p_{p}\right):p_{s}={\left(2/F_{1,0}-1/p_{p}\right)}^{-1},{\left(2/F_{1,0}-1\right)}^{-1}\leq p_{p}\leq 1\}$. If $p_{v}\left(f_{\mathrm{obs}};F_{1,0}\right)<\alpha^{*}$, then we reject $H_{0}:F_{1}=F_{1,0}$, where $\alpha^{*}$ is a pre‐specified significance level. It is worthy to note that the *p* value defined in Formula (5) is larger than the *p* value in Formula (4), provided that $F_{1}\left(p_{s0},p_{p0}\right)=F_{1,0}$. This implies rejecting $H_{0}:F_{1}=F_{1,0}$ is more challenging than rejecting $H_{0}:F_{1}=F_{1}\left(p_{s0},p_{p0}\right)$.

#### 2.1.3 Power and Sample Size Calculation

Given N, S, and a pre‐specified significance level of $\alpha^{*}$, the power to detect whether a classifier's $F_{1}$ score differs from a target $F_{1}$ score defined by a specified sensitivity $p_{s0}$ and precision $p_{p0}$ is given by

(6) $$\sum_{f\in \left\{f:p_{v}\left(f;p_{s0},\;p_{p0}\right)<\alpha^{*}\right\}}g\left(f;p_{s1},p_{p1}\right),$$

when the classifier has a sensitivity $p_{s1}$ and precision $p_{p1}$. The powers for situations in which the target $F_{1}$ score is specified without given $p_{s0}$ and $p_{p0}$ are presented in the Supporting Information. To facilitate the calculation of N and S required to achieve a desired power, we set $S=Np_{I}$ and increase N, where $p_{I}$ is a pre‐specified ratio of S/N. For example, $p_{I}$ can be the proportion of positive instances.

### 2.2 Methods for Comparing $F_{1}$ Scores Between Two Classifiers

We assume that a common dataset is used to compare the $F_{1}$ scores of two classifiers, and that the predictions made by the two classifiers are independent. Let the estimated $F_{1}$ scores of classifiers 1 and 2 be denoted by $f_{1,1}=2d_{1}/\left(b_{1}+d_{1}+S\right)$ and $f_{1,2}=2d_{2}/\left(b_{2}+d_{2}+S\right)$, where $b_{k}$ and $d_{k}$ are the number of false positives and true positives determined by classifier k (k = 1, 2) and are assumed to be independent. The difference in the estimated $F_{1}$ scores between the two classifiers is denoted as $\Delta f_{1}=f_{1,1}-f_{1,2}$.

#### 2.2.1 Confidence Interval

Conditional on S and N−S, the probability mass function of $\Delta f_{1}=\Delta f$ can be expressed as follows:

(7) $$h\left(\Delta f;p_{s1},p_{p1},p_{s2},p_{p2}\right)=\sum_{\left(f_{1,1},f_{1,2}\right)\in Q_{2}\left(\Delta f\right)}g\left(f_{1,1};p_{s1},p_{p1}\right)g\left(f_{1,2};p_{s2},p_{p2}\right),$$

where $Q_{2}\left(\Delta f\right)=\{\left(f_{1,1},f_{1,2}\right):f_{1,1}-f_{1,2}=\Delta f,f_{1,1}\in {ℱ}_{N,S},f_{1,2}\in {ℱ}_{N,S}\}$ and $\Delta f\in {\Delta ℱ}_{N,S}=\{f_{1,1}-f_{1,2}:f_{1,1}\in {ℱ}_{N,S},f_{1,2}\in {ℱ}_{N,S}\}$. The function g is defined in Formula (2). Therefore, a 100($1-\alpha^{*}$)% confidence interval for the difference in $F_{1}$ scores can be constructed, given observed values $b_{k}=b_{\mathrm{obs},k}$ and $d_{k}=d_{\mathrm{obs},k}$, k = 1 and 2, where $1-\alpha^{*}$ is a pre‐specified confidence level. This interval estimate for $\Delta f_{1}$ is denoted as [L,U], where.

(8) $$\begin{array}{cc} & L=\max\left\{l\in {\Delta ℱ}_{N,S}:\sum_{\Delta f\leq l}h\left(\Delta f;{\tilde{p}}_{s1},{\tilde{p}}_{p1},{\tilde{p}}_{s2},{\tilde{p}}_{p2}\right)\leq \alpha^{*}/2\right\}\;\;\text{and}\;\\ & \;U=\min\left\{u\in {\Delta ℱ}_{N,S}:\sum_{u\leq \Delta f}h\left(\Delta f;{\tilde{p}}_{s1},{\tilde{p}}_{p1},{\tilde{p}}_{s2},{\tilde{p}}_{p2}\right)\leq \alpha^{*}/2\right\}. \end{array}$$

Similar to Section 2.1, to address slightly insufficient coverage probability in confidence interval estimation when N and S are small, we set $b_{\mathrm{obs},k}$ = 0.5 if $b_{\mathrm{obs},k}$ = 0, and consider ${\tilde{p}}_{\mathrm{sk}}=(d_{\mathrm{obs},k}+0.5)/\left(S+1\right)$ and ${\tilde{p}}_{\mathrm{pk}}=(d_{\mathrm{obs},k}+0.5)/(b_{\mathrm{obs},k}+d_{\mathrm{obs},k}+1)$, k = 1 and 2.

#### 2.2.2 Hypothesis Testing

To test $H_{0}:F_{1,1}=F_{1,2}$ vs. $H_{1}:F_{1,1}\neq F_{1,2}$, we consider two ways of constructing the null distribution of $\Delta f_{1}$: assume (i) $F_{1,1}=F_{1,2}={\hat{F}}_{1,0}$, where ${\hat{F}}_{1,0}$ = $F_{1}\left({\bar{p}}_{s},{\bar{p}}_{p}\right)$ with ${\bar{p}}_{s}$ and $\;{\bar{p}}_{p}$ being defined in the subsequent section, and (ii) $F_{1,1}=F_{1,2}=F_{1,0}$ for all possible $F_{1,0}$ without specifying precision and sensitivity values.

##### 2.2.2.1 $F_{1,1}=F_{1,2}={\hat{F}}_{1,0}$

We consider ${\hat{F}}_{1,0}$ = $F_{1}\left({\bar{p}}_{s},{\bar{p}}_{p}\right)$, where ${\bar{p}}_{s}=\left({\hat{p}}_{s1}+{\hat{p}}_{s2}\right)/2$ and ${\bar{p}}_{p}=\left({\hat{p}}_{p1}+{\hat{p}}_{p2}\right)/2$. Here, ${\hat{p}}_{\mathrm{sk}}=d_{\mathrm{obs},k}/S$ and ${\hat{p}}_{\mathrm{pk}}=d_{\mathrm{obs},k}/\left(b_{\mathrm{obs},k}+d_{\mathrm{obs},k}\right)$, k = 1 and 2. Therefore, the null distribution of ${\Delta f}_{1}=\Delta f$ can be expressed as $h\left(\Delta f;{\bar{p}}_{s},{\bar{p}}_{p},{\bar{p}}_{s},{\bar{p}}_{p}\right)$, and the *p* value given $\Delta f_{\mathrm{obs}}=f_{\mathrm{obs},1}-f_{\mathrm{obs},2}$ is given by

(9) $$p_{v2}\left(\Delta f_{\mathrm{obs}};{\bar{p}}_{s},{\bar{p}}_{p}\right)=\sum_{\Delta f\in R_{2}}h\left(\Delta f;{\bar{p}}_{s},{\bar{p}}_{p},{\bar{p}}_{s},{\bar{p}}_{p}\right),$$

where $f_{\mathrm{obs},k}=2d_{\mathrm{obs},k}/\left(b_{\mathrm{obs},k}+d_{\mathrm{obs},k}+S\right)$, $R_{2}=\{\Delta f:C_{2}\left(\Delta f\right)\leq C_{2}\left({\Delta f}_{\mathrm{obs}}\right)\;\}$, and $C_{2}\left(u\right)=\min\{\sum_{\Delta f\leq u}h\left(f;{\bar{p}}_{s},{\bar{p}}_{p},{\bar{p}}_{s},{\bar{p}}_{p}\right),\sum_{\Delta f\geq u}h\left(f;{\bar{p}}_{s},{\bar{p}}_{p},{\bar{p}}_{s},{\bar{p}}_{p}\right)\}$. $R_{2}$ is the region of that Δf is more extreme than ${\Delta f}_{\mathrm{obs}}$. If $p_{v2}\left({\Delta f}_{\mathrm{obs}};{\bar{p}}_{s},{\bar{p}}_{p}\right)<\alpha^{*}$, we reject $H_{0}:F_{1,1}=F_{1,2}={\hat{F}}_{1,0}$, where $\alpha^{*}$ is a pre‐specified significance level. The *p* values for one‐sided hypothesis tests are presented in the Supporting Information.

##### 2.2.2.2 $F_{1,1}=F_{1,2}=F_{1,0}$ for All Possible $F_{1,0}$

The *p* value given $\Delta f_{\mathrm{obs}}$ is given by

(10) $$p_{v2}\left(\Delta f_{\mathrm{obs}}\right)=\underset{0<F_{1,0}<1}{\sup}p_{v2}\left(\Delta f_{\mathrm{obs}};F_{1,0}\right),$$

where $p_{v2}\left(\Delta f_{\mathrm{obs}};F_{1,0}\right)=\underset{\left(p_{s},p_{p}\right)\in \Theta \left(F_{1,0}\right)}{\sup}p_{v2}\left(\Delta f_{\mathrm{obs}};p_{s},p_{p}\right)$ and $\Theta \left(F_{1,0}\right)$ is defined in Section 2.1.2.2. However, using the *p* value may be too conservative. We refine the search region of $F_{1,0}$ by replacing the interval (0,1) with the 2.5th and 97.5th percentiles of the distribution $g\left(f;{\bar{p}}_{s},{\bar{p}}_{p}\right)$. The function g is defined in Formula (2) of Section 2.1.1.

#### 2.2.3 Power and Sample Size Calculation

Given N, S, and a pre‐specified significance level of $\alpha^{*}$, the power to detect the difference between $F_{1}(p_{s1}$, $p_{p1})$ and $F_{1}(p_{s2}$, $p_{p2})$ is given by

(11) $$\sum_{\Delta f\in \left\{\Delta f:p_{v2}\left(\Delta f;{\bar{p}}_{s},{\bar{p}}_{p}\right)<\alpha^{*}\right\}}h\left(\Delta f;p_{s1},p_{p1},p_{s2},p_{p2}\right),$$

where ${\bar{p}}_{s}=\left(p_{s1}+p_{s2}\right)/2$ and ${\bar{p}}_{p}=\left(p_{p1}+p_{p2}\right)/2$. The power for $F_{1,1}$ and $F_{1,2}$ in which the sensitivities and precisions are not specified is presented in the Supporting Information. Based on these power formulas, the required N and S to achieve a desired statistical power at the significance level of $\alpha^{*}$ can be calculated.

### 2.3 Normal Approximation

When N and S are large, calculating *p* value and power using exact distributions of $f_{1}$ and $∆f_{1}$ can be computationally intensive. To improve computational efficiency in such cases, we use normal approximations for the distributions of $f_{1}$ and $∆f_{1}$. Below, we demonstrate that the distributions of $f_{1}$ and $∆f_{1}$ asymptotically approximate normal distributions.

In Section 2.1, we assume that b and d follow binomial distributions $\mathrm{Bin}\;\left(N-S,p_{\alpha }\right)$ and $\mathrm{Bin}\left(S,p_{s}\right)$. Given that N−S and S are large, by the central limit theorem, b/S (= b/(N−S)×(N−S)/S) and d/S approximate

(12) $$N_{1}\left(\frac{\left(N-S\right)}{S}\;p_{\alpha },\frac{\left(N-S\right)}{S^{2}}p_{\alpha }\left(1-p_{\alpha }\right)\right)\;\;\text{and}\;N_{1}\left(p_{s},\frac{1}{S}p_{s}\left(1-p_{s}\right)\right).$$

Since b and d are independent, we can further express (12) as:

(13) $$\sqrt{S}\left(\hat{\mu }-\mu \right)\;\text{approximates}\;N_{2}\left(0,\sum \right),$$

where $\hat{\mu }={\left(\frac{b}{S},\frac{d}{S}\right)}^{T}$, $\mu ={\left(\frac{N-S}{S}p_{\alpha },p_{s}\right)}^{T}$, and

$$\sum =\left(\begin{array}{ll} \frac{\left(N-S\right)}{S}p_{\alpha }\left(1-p_{\alpha }\right) & 0\\ 0 & p_{s}\left(1-p_{s}\right) \end{array}\right).$$

Let $w\left({\left(x,y\right)}^{T}\right)=2y/\left(x+y+1\right)$ and 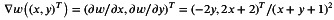. Then, using the Delta method, $\sqrt{S}\left(w\left(\hat{\mu }\right)-w\left(\mu \right)\right)$ approximates 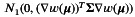. Therefore, $f_{1}=w\left(\hat{\mu }\right)=2d/\left(b+d+S\right)$ is shown to approximate a normal distribution with the expected value $E\left(f_{1}\right)\approx w\left(\mu \right)=2p_{s}/\left(\frac{N-S}{S}p_{\alpha }+p_{s}+1\right)$. An approximation for $p_{\alpha }$ can be derived as: $p_{\alpha }\approx \frac{S}{N-S}\left\{p_{s}\left(2{\left(E\left(f_{1}\right)\right)}^{-1}-1\right)-1\right\}$. Furthermore, the asymptotic normality of $∆f_{1}$ follows directly from the fact that the difference between two independent normally distributed variables is itself normally distributed.

However, N and S required for the normal approximation to be accurate depend on the underlying $p_{s}$ and $p_{p}$. To facilitate the usage, psF1 automatically determines whether to use the normal approximation based on the number of mass points in the $f_{1}$ distribution. The number of mass points serves as a practical proxy for the effective sample size and discreteness of the underlying true‐positive and false‐positive distributions. When this number is large, these distributions are well approximated by normal distributions via the central limit theorem, and the resulting $f_{1}$ distribution becomes sufficiently smooth for the normal approximation to be accurate. Simulation studies show that results obtained using this automatic selection procedure are very close to those based on the exact distribution (Tables S1 and S2).

### 2.4 Extension to the $F_{\beta }$ Score

The proposed methods can be straightforwardly extended to the $F_{\beta }$ score which is defined as:

$$F_{\beta }=F_{\beta }\left(p_{s},p_{p}\right)={\left(\frac{p_{p}^{-1}+\beta^{2}p_{s}^{-1}}{1+\beta^{2}}\right)}^{-1}=\frac{\left(1+\beta^{2}\right)p_{p}p_{s}}{\beta^{2}p_{p}+p_{s}}.$$

When β = 1, the $F_{\beta }$ score reduces to the $F_{1}$ score. For β < 1, the metric places greater emphasis on precision, while for β > 1, it gives more weight to sensitivity. The procedures for constructing confidence intervals, conducting hypothesis testing, and performing power and sample size calculations for $F_{\beta }$ scores are similar to those developed for $F_{1}$ scores. Specifically, we replace $f_{1}$, $∆f_{1}$, $F_{1,0}$, $F_{1,1}$, and $F_{1,2}$ with $f_{\beta }$, $∆f_{\beta }$, $F_{\beta ,0}$, $F_{\beta ,1}$, and $F_{\beta ,2}$, and adjust $p_{\alpha }$ to satisfy $E\left(f_{\beta }\right|\;S,N-S,p_{s},p_{\alpha })=F_{\beta }$, that is,

(14) $$\sum_{d=0}^{S}\sum_{b=0}^{N-S}\frac{\left(1+\beta^{2}\right)d}{b+d+\beta^{2}S}\mathrm{Bin}\left(b;N-S,p_{\alpha }\right)\mathrm{Bin}\left(d;S,p_{s}\right)=F_{\beta },$$

where $f_{\beta }=\left(1+\beta^{2}\right)d/\left(d+b+S\beta^{2}\right)$ serves as the estimate for $F_{\beta }$.

## 3 Simulation Studies

This section consists of three subsections. First, we compared the proposed method psF1‐CI with existing CI methods. Second, we evaluated the proposed hypothesis testing methods psF1 and psF1‐CI against adapted CI‐based tests, where the null hypothesis is rejected if the CI does not include the target $F_{1}$ score. Third, we assessed the statistical power of psF1 through simulation.

### 3.1 Performance Comparison of Interval Estimation Methods

We compared the performance of psF1‐CI with four CI methods, including CP (Clopper–Pearson method), Wald (Wald‐type method), WD (Wilson direct method), and WID (Wilson indirect method), in terms of interval length and coverage probability. When the target coverage probability is met, a shorter interval indicates greater precision.

#### 3.1.1 Interval Estimation of the $F_{1}$ Score From a Single Classifier

We considered three scenarios that yield the same $F_{1}$ score but differ in the underlying tradeoffs between sensitivity and precision: (i) high sensitivity and low precision ($p_{p1}$ = 0.6 and $p_{s1}$ = 0.9); (ii) high precision and low sensitivity ($p_{p1}$ = 0.9 and $p_{s1}$ = 0.6); (iii) balanced precision and sensitivity ($p_{p1}$ = $p_{s1}$ = 0.72). Each scenario was evaluated under four configurations with varying sample sizes and numbers of actual positives: (N, S) = (150, 60), (100, 40), (50, 20), and (1000, 20). In the first scenario, psF1‐CI produced noticeably shorter intervals than the other methods, indicating greater estimation precision (Table 3). In the second scenario, the WID method yielded the shortest interval, though its advantage was marginal. In the third scenario with balanced precision and sensitivity, both psF1‐CI and WID showed superior performance. Overall, psF1‐CI outperformed the four methods across different settings and demonstrated robust performance. In contrast, CP, Wald, WD, and WID, which condition on the sum of predicted positives and false negatives, appeared more vulnerable to the scenario with high sensitivity but low precision. Additional scenarios involving more extreme tradeoffs between sensitivity and precision, as well as greater class imbalance, yielded similar results (Tables S3 and S4). Notably, in settings with pronounced sensitivity–precision imbalance, psF1‐CI produced substantially shorter confidence intervals in certain cases characterized by high sensitivity and low precision, approximately half the length of those obtained using other methods.

**TABLE 3:** Mean (standard deviation) of the interval length of 95% CI and coverage probability (shown in the second rows) across 1000 simulation runs for the $F_{1}$ score of a single classifier.

$p_{p1}$ = 0.6, $p_{s1}$ = 0.9 ($F_{1,1}$ = 0.720)					
	psF1‐CI	CP	Wald	WD	WID
*N* = 150, *S* = 60	0.120 (0.010) 0.943	0.170 (0.007) 0.999	0.162 (0.007) 0.993	0.163 (0.006) 0.996	0.160 (0.006) 0.997
*N* = 100, *S* = 40	0.148 (0.015) 0.951	0.209 (0.010) 0.995	0.199 (0.010) 0.989	0.201 (0.009) 0.990	0.195 (0.010) 0.987
*N* = 50, *S* = 20	0.219 (0.029) 0.959	0.300 (0.020) 0.995	0.280 (0.021) 0.987	0.285 (0.016) 0.989	0.270 (0.018) 0.991
*N* = 1000, *S* = 20	0.248 (0.023) 0.961	0.299 (0.021) 0.985	0.279 (0.022) 0.972	0.284 (0.017) 0.970	0.269 (0.019) 0.974

$p_{p1}$ = 0.9, $p_{s1}$ = 0.6 ($F_{1,1}$ = 0.720)					
	psF1‐CI	CP	Wald	WD	WID
*N* = 150, *S* = 60	0.202 (0.018) 0.943	0.210 (0.019) 0.954	0.199 (0.018) 0.940	0.201 (0.017) 0.945	0.195 (0.017) 0.943
*N* = 100, *S* = 40	0.248 (0.026) 0.952	0.259 (0.027) 0.965	0.243 (0.028) 0.942	0.246 (0.024) 0.947	0.237 (0.025) 0.949
*N* = 50, *S* = 20	0.358 (0.050) 0.962	0.369 (0.052) 0.961	0.342 (0.055) 0.932	0.347 (0.040) 0.948	0.324 (0.045) 0.952
*N* = 1000, *S* = 20	0.358 (0.050) 0.963	0.369 (0.052) 0.961	0.342 (0.056) 0.929	0.347 (0.040) 0.949	0.324 (0.045) 0.952

$p_{p1}$ = 0.72, $p_{s1}$ = 0.72 ($F_{1,1}$ = 0.720)					
	psF1‐CI	CP	Wald	WD	WID
*N* = 150, *S* = 60	0.172 (0.014) 0.954	0.191 (0.013) 0.973	0.182 (0.013) 0.956	0.183 (0.012) 0.962	0.179 (0.012) 0.966
*N* = 100, *S* = 40	0.211 (0.021) 0.954	0.235 (0.020) 0.970	0.222 (0.020) 0.949	0.225 (0.018) 0.967	0.217 (0.018) 0.963
*N* = 50, *S* = 20	0.306 (0.041) 0.953	0.335 (0.038) 0.970	0.312 (0.040) 0.943	0.317 (0.031) 0.957	0.299 (0.034) 0.963
*N* = 1000, *S* = 20	0.313 (0.039) 0.958	0.335 (0.039) 0.968	0.312 (0.041) 0.936	0.317 (0.031) 0.953	0.298 (0.034) 0.956

#### 3.1.2 Interval Estimation of Differences in $F_{1}$ Scores Between Two Classifiers

Since no established methods exist for constructing confidence intervals for differences in $F_{1}$ scores, we focused exclusively on evaluating the interval estimation performance of psF1‐CI. Three scenarios were considered, where classifier 2 has: (i) higher precision and sensitivity, (ii) higher precision but the same sensitivity, and (iii) the same precision but higher sensitivity, compared with classifier 1. The corresponding parameters for the three scenarios are: (i) ($p_{p2}$ = 0.6, $p_{s2}$ = 0.9) vs. ($p_{p1}$ = 0.5, $p_{s1}$ = 0.8), (ii) ($p_{p2}$ = 0.6, $p_{s2}$ = 0.9) vs. ($p_{p1}$ = 0.5, $p_{s1}$ = 0.9), and (iii) ($p_{p2}$ = 0.9, $p_{s2}$ = 0.9) vs. ($p_{p1}$ = 0.9, $p_{s1}$ = 0.7), Each scenario was evaluated under four configurations: (N, S) = (250, 100), (100, 40), (50, 20), and (1000, 20). The psF1‐CI method consistently achieved the target coverage probability of 0.95 across all settings. However, it tended to be slightly conservative when S was small, particularly when S = 20 (Table S5).

### 3.2 Performance Comparison of Hypothesis Testing Methods

#### 3.2.1 Hypothesis Testing of the $F_{1}$ Score From a Single Classifier

We compared the statistical power of psF1 with several CI‐ and CrI‐based methods, including psF1‐CI, CP, Wald, WD, WID, and Wang [12]. Although these methods were originally designed for interval estimation rather than hypothesis testing, they can be adapted for testing by rejecting the null hypothesis if the interval estimates do not include the null $F_{1}$ value. We evaluated the performance under three scenarios: the new classifier has (i) higher precision and sensitivity ($p_{p1}$ = 0.6, $p_{s1}$ = 0.9) versus ($p_{p0}$ = 0.5, $p_{s0}$ = 0.8), (ii) higher precision but the same sensitivity ($p_{p1}$ = 0.6, $p_{s1}$ = 0.9) versus ($p_{p0}$ = 0.5, $p_{s0}$ = 0.9), and (iii) equal precision but higher sensitivity ($p_{p1}$ = 0.9, $p_{s1}$ = 0.9) versus ($p_{p0}$ = 0.9, $p_{s0}$ = 0.7). Each scenario was tested under four configurations: (N, S) = (150, 60), (100, 40), (50, 20), and (1000, 20). In the first two scenarios, psF1 and psF1‐CI outperformed other methods in terms of power (Table 4). In the third scenario, the Wald method showed the highest power, followed by psF1‐CI, while psF1 and WID performed comparably, and CP, WD, and Wang showed the lowest power. However, it is important to note that in third scenario, the Wald method exhibited inflated type I error rates (Table S6).

**TABLE 4:** Power comparison between methods for the $F_{1}$ score of a single classifier at the significance level of 0.05.

$p_{p0}$ = 0.5, $p_{s0}$ = 0.8 ($F_{1,0}$ = 0.615); $p_{p1}$ = 0.6, $p_{s1}$ = 0.9 ($F_{1,1}$ = 0.720)							
	psF1	psF1‐CI	CP	Wald	WD	WID	Wang^a^
*N* = 150, *S* = 60	0.916	0.903	0.662	0.763	0.662	0.714	0.662
*N* = 100, *S* = 40	0.765	0.751	0.386	0.521	0.386	0.448	0.386
*N* = 50, *S* = 20	0.434	0.393	0.100	0.274	0.100	0.168	0.100
*N* = 1000, *S* = 20	0.327	0.335	0.135	0.310	0.134	0.203	0.134

$p_{p0}$ = 0.5, $p_{s0}$ = 0.9 ($F_{1,0}$ = 0.643); $p_{p1}$ = 0.6, $p_{s1}$ = 0.9 ($F_{1,1}$ = 0.720)							
	psF1	psF1‐CI	CP	Wald	WD	WID	Wang^a^
*N* = 150, *S* = 60	0.841	0.685	0.319	0.459	0.286	0.356	0.301
*N* = 100, *S* = 40	0.674	0.470	0.157	0.311	0.144	0.196	0.148
*N* = 50, *S* = 20	0.425	0.249	0.040	0.153	0.033	0.064	0.034
*N* = 1000, *S* = 20	0.240	0.210	0.070	0.188	0.047	0.093	0.056

$p_{p0}$ = 0.9, $p_{s0}$ = 0.7 ($F_{1,0}$ = 0.788); $p_{p1}$ = 0.9, $p_{s1}$ = 0.9 ($F_{1,1}$ = 0.9)							
	psF1	psF1‐CI	CP	Wald	WD	WID	Wang^a^
*N* = 150, *S* = 60	0.860	0.895	0.852	0.927	0.840	0.876	0.836
*N* = 100, *S* = 40	0.720	0.761	0.661	0.839	0.615	0.711	0.602
*N* = 50, *S* = 20	0.382	0.453	0.318	0.614	0.239	0.382	0.239
*N* = 1000, *S* = 20	0.380	0.390	0.316	0.611	0.238	0.380	0.238

^a^ Power: The probability that CrI does not include the null $F_{1}$ value.

#### 3.2.2 Hypothesis Testing of Differences in $F_{1}$ Scores Between Two Classifiers

We evaluated the statistical power of psF1 in comparison with psF1‐CI, permutation, G&G [11], and Takahashi [16] (the covariance part of the test statistics is set as zero). The parameter settings were consistent with those described in Section 3.1. In the first two scenarios, psF1 and the permutation approach achieved the highest power, followed by psF1‐CI, while the G&G and Takahashi approaches obtained substantially lower power (Table 5). In the third scenario, all methods performed similarly, with psF1 showing slightly better power. Across three scenarios, type I error rates were well controlled for all methods (Table S7). Additional scenarios with greater class imbalance in the power comparison obtained similar results (Table S8), with psF1 achieving the highest statistical power.

**TABLE 5:** Power comparison between methods for the difference between two classifiers' $F_{1}$ scores at the significance level of 0.05.

$p_{p1}$ = 0.5, $p_{s1}$ = 0.8 ($F_{1,1}$ = 0.615); $p_{p2}$ = 0.6, $p_{s2}$ = 0.9 ($F_{1,2}$ = 0.720)					
	psF1	psF1‐CI	Permutation	G&G^a^	Takahashi
*N* = 250, *S* = 100	0.865	0.855	0.850	0.599	0.612
*N* = 100, *S* = 40	0.465	0.446	0.446	0.192	0.222
*N* = 50, *S* = 20	0.259	0.210	0.246	0.059	0.080
*N* = 1000, *S* = 20	0.192	0.155	0.193	0.090	0.115

$p_{p1}$ = 0.5, $p_{s1}$ = 0.9 ($F_{1,1}$= 0.643); $p_{p2}$ = 0.6, $p_{s2}$ = 0.9 ($F_{1,2}$ = 0.720)					
	psF1	psF1‐CI	Permutation	G&G^a^	Takahashi
*N* = 250, *S* = 100	0.719	0.711	0.684	0.334	0.349
*N* = 100, *S* = 40	0.362	0.329	0.386	0.067	0.084
*N* = 50, *S* = 20	0.194	0.151	0.203	0.020	0.035
*N* = 1000, *S* = 20	0.156	0.099	0.141	0.040	0.083

$p_{p1}$ = 0.9, $p_{s1}$ = 0.7 ($F_{1,1}$ = 0.788); $p_{p2}$ = 0.9, $p_{s2}$ = 0.9 ($F_{1,2}$ = 0.9)					
	psF1	psF1‐CI	Permutation	G&G^a^	Takahashi
*N* = 250, *S* = 100	0.825	0.807	0.828	0.803	0.804
*N* = 100, *S* = 40	0.441	0.415	0.430	0.395	0.414
*N* = 50, *S* = 20	0.236	0.188	0.210	0.179	0.224
*N* = 1000, *S* = 20	0.230	0.192	0.198	0.183	0.226

^a^ Power: The probability that CrI does not include zero.

### 3.3 Validation for Power Calculation via Simulation

#### 3.3.1 Power Calculation of the $F_{1}$ Score From a Single Classifier

We validated the statistical power estimated by psF1 to determine whether a new classifier's $F_{1}$ score, defined by precision $p_{p1}$ and sensitivity $p_{s1}$, differs from a target $F_{1}$ score defined by precision $p_{p0}$ and sensitivity $p_{s0}$. The power estimates were compared against empirical powers obtained from 1000 simulation runs, using the same scenario settings as in Section 3.2. In addition to the $F_{1}$ score, we also evaluated scenarios using the $F_{\beta }$ score with β = 0.5 and 2. The validation results demonstrated that the power estimates closely matched those from the simulations (Table 6).

**TABLE 6:** Empirical power (estimated power by psF1) for the $F_{\beta }$ score of a single classifier at the significance level of 0.05.

$p_{p0}$ = 0.5, $p_{s0}$ = 0.8; $p_{p1}$ = 0.6, $p_{s1}$ = 0.9			
	β = 0.5	β = 1	β = 2
*N* = 150, *S* = 60	0.913 (0.910)	0.916 (0.913)	0.831 (0.818)
*N* = 100, *S* = 40	0.774 (0.765)	0.765 (0.762)	0.621 (0.619)
*N* = 50, *S* = 20	0.421 (0.433)	0.434 (0.460)	0.357 (0.366)
*N* = 1000, *S* = 20	0.256 (0.256)	0.327 (0.324)	0.313 (0.313)

$p_{p0}$ = 0.5, $p_{s0}$ = 0.9; $p_{p1}$ = 0.6, $p_{s1}$ = 0.9			
	β = 0.5	β = 1	β = 2
*N* = 150, *S* = 60	0.918 (0.920)	0.841 (0.825)	0.332 (0.347)
*N* = 100, *S* = 40	0.793 (0.788)	0.674 (0.677)	0.307 (0.306)
*N* = 50, *S* = 20	0.523 (0.538)	0.425 (0.447)	0.236 (0.240)
*N* = 1000, *S* = 20	0.274 (0.285)	0.240 (0.240)	0.149 (0.157)

$p_{p0}$ = 0.9, $p_{s0}$ = 0.7; $p_{p1}$ = 0.9, $p_{s1}$ = 0.9			
	β = 0.5	β = 1	β = 2
*N* = 150, *S* = 60	0.287 (0.280)	0.860 (0.874)	0.970 (0.974)
*N* = 100, *S* = 40	0.234 (0.233)	0.720 (0.718)	0.851 (0.880)
*N* = 50, *S* = 20	0.126 (0.137)	0.382 (0.403)	0.495 (0.509)
*N* = 1000, *S* = 20	0.133 (0.147)	0.380 (0.403)	0.495 (0.511)

#### 3.3.2 Power Calculation of Differences in $F_{1}$ Scores Between Two Classifiers

We validated the statistical power estimated by psF1 to detect differences between two classifiers' $F_{1}$ scores by comparing the estimated powers against empirical results from 1000 simulation runs. The scenario settings were the same as those described in Section 3.1. The estimated powers were similar to the empirical powers from the simulations (Table 7).

**TABLE 7:** Empirical power (estimated power by psF1) for the difference between two classifiers' $F_{\beta }$ scores at the significance level of 0.05.

$p_{p1}$ = 0.5, $p_{s1}$= 0.8; $p_{p2}$ = 0.6, $p_{s2}$ = 0.9			
	β = 0.5	β = 1	β = 2
*N* = 250, *S* = 100	0.860 (0.851)	0.865 (0.850)	0.734 (0.729)
*N* = 100, *S* = 40	0.463 (0.467)	0.465 (0.466)	0.361 (0.361)
*N* = 50, *S* = 20	0.256 (0.258)	0.259 (0.258)	0.196 (0.201)
*N* = 1000, *S* = 20	0.165 (0.184)	0.192 (0.206)	0.179 (0.195)

$p_{p1}$ = 0.5, $p_{s1}$ = 0.9; $p_{p2}$ = 0.6, $p_{s2}$ = 0.9			
	β = 0.5	β = 1	β = 2
*N* = 250, *S* = 100	0.860 (0.855)	0.719 (0.703)	0.277 (0.236)
*N* = 100, *S* = 40	0.467 (0.471)	0.362 (0.343)	0.135 (0.119)
*N* = 50, *S* = 20	0.254 (0.257)	0.194 (0.192)	0.097 (0.083)
*N* = 1000, *S* = 20	0.155 (0.172)	0.156 (0.146)	0.065 (0.082)

$p_{p1}$ = 0.9, $p_{s1}$ = 0.7; $p_{p2}$ = 0.9, $p_{s2}$ = 0.9			
	β = 0.5	β = 1	β = 2
*N* = 250, *S* = 100	0.263 (0.267)	0.825 (0.813)	0.936 (0.930)
*N* = 100, *S* = 40	0.135 (0.138)	0.441 (0.430)	0.589 (0.574)
*N* = 50, *S* = 20	0.079 (0.097)	0.236 (0.237)	0.313 (0.311)
*N* = 1000, *S* = 20	0.086 (0.097)	0.230 (0.235)	0.311 (0.311)

## 4 Real Application

This section illustrates the application of psF1 through two real cases: the detection of Trisomy 21 and the prediction of postoperative urinary retention.

### 4.1 Application 1: The Detection of Trisomy 21 (Down's Syndrome)

Trisomy 21 leads to intellectual disability and developmental delays in children. Among high‐risk women, cell‐free DNA (cfDNA) testing has been shown to be a more effective diagnostic test for fetal trisomy compared to standard screening. In the large prospective NEXT study (Noninvasive Examination of Trisomy), Norton et al. [7] evaluated whether cfDNA testing also outperforms standard screening during the first trimester in a general prenatal population. The study enrolled 18 955 pregnant women undergoing first‐trimester aneuploidy screening between March 2012 and April 2013. Of these, 15 841 pregnancies were included in the final analysis, with only 38 cases of trisomy 21, creating a highly imbalanced dataset. Performance was assessed using AUC, with cfDNA testing demonstrating significantly higher accuracy (AUC = 0.999) compared with standard screening (AUC = 0.958; *p* = 0.001).

In addition to AUC, the study reported sensitivity, specificity and PPV for both diagnostic tests. cfDNA testing demonstrated outstanding performance, with 100% sensitivity, 99.9% specificity, and a PPV of 80.9%, corresponding to 38 true positives (TPs) and 9 false positives (FPs), yielding an overall accuracy of 99.9%. In contrast, standard screening showed substantially lower performance, with 78.9% sensitivity, 94.6% specificity, and a PPV of only 3.4%, corresponding to 30 TPs and 854 FPs, and an overall accuracy of 94.6% (Table S9). Based on these results, the estimated $F_{1}$ scores were 0.894 for cfDNA testing and 0.065 for standard screening, showing a substantial difference of 0.829, which far exceeds the AUC difference (0.041) and the accuracy difference (0.053). This highlights that F1 score is a more sensitive metric in class‐imbalanced settings. Using the proposed psF1, the *p* value for the estimated $F_{1}$ difference was far less than 0.001, with a 95% CI of (0.751, 0.883). This suggests that a much smaller sample size would be sufficient to detect the $F_{1}$ score difference. Given the observed PPV and sensitivity values, enrolling only 1584 pregnant women and 4 cases of trisomy 21 (approximately one‐tenth of the original sample size) would provide 98% power to detect the 0.829 difference in $F_{1}$ scores at a two‐sided significance level of 0.001. In practical terms, this corresponds to a 98% probability of concluding that cfDNA testing is more effective than standard screening based on a *p* < 0.001.

We also performed statistical inference for the individual F1 scores of both tests. For cfDNA testing, the 95% confidence intervals using psF1‐CI, CP, Wald, WD, and WID were (0.817, 0.950), (0.801, 0.952), (0.825, 0.963), (0.796, 0.943), and (0.806, 0.945), respectively, with corresponding interval lengths of 0.133, 0.152, 0.138, 0.147, and 0.139. For standard screening, the 95% confidence intervals were (0.054, 0.077), (0.046, 0.091), (0.043, 0.087), (0.046, 0.090), and (0.046, 0.091), with interval lengths of 0.023, 0.046, 0.044, 0.044, and 0.045. For reference, the 95% CrIs estimated by Wang's Bayesian approach were (0.793, 0.938) with a length of 0.145 for cfDNA testing and (0.046, 0.091) with a length of 0.045 for standard screening. psF1‐CI produced the shortest interval, especially for standard screening, where the interval length was approximately half that of the other methods, indicating greater precision compared to alternative methods.

### 4.2 Application 2: Prediction of Postoperative Urinary Retention (POUR)

Porche et al. [17] proposed three predictive models for POUR using data collected from 1311 adult patients who underwent lumbar spine surgery at the University of Florida between June 1, 2017, and June 1, 2019, and evaluated their performance based on AUC. In this imbalanced set containing more non‐POUR than POUR patients, the differences in AUCs between models were less obvious than those observed in $F_{1}$ scores. Thus, comparing $F_{1}$ scores is more effective. This application demonstrates the advantages of psF1: higher statistical powers for detecting the $F_{1}$ score difference and more precise interval estimates. The details of the application are provided in the Supporting Information.

## 5 Discussion

In this study, we propose psF1, a unified statistical framework for interval estimation, hypothesis testing, and power and sample size calculation for both single and comparative $F_{1}$ and $F_{\beta }$ scores. psF1 is particularly advantageous for evaluating classifiers under high imbalance data, a setting common in medical applications. Metrics such as accuracy and AUC can be dominated by high specificity when the proportion of negatives is large. Consequently, classifiers with low precision or low sensitivity may still achieve accuracy and AUC values comparable to those of better‐performing classifiers. These small performance differences make meaningful comparisons challenging and often require large sample sizes when study planning is based on accuracy or AUC. In contrast, low sensitivity or low precision directly yields a low $F_{1}$ score, leading to clearer performance distinction. Therefore, $F_{1}$‐based approaches such as psF1 may, in certain imbalanced settings, provide greater power for performance evaluation and potentially require fewer samples for study planning than accuracy‐ or AUC‐based approaches, as demonstrated in Real Application 1.

Unlike existing approaches which employ simulation‐dependent Bayesian methods and non‐parametric permutation, psF1 is based on the exact probability distributions of the estimated $F_{1}$ scores, avoiding time‐consuming simulations. Although computing exact distributions can be computationally intensive for large samples, we demonstrate that the exact distributions asymptotically approximate normal distributions as sample sizes increase, thereby reducing computational burden and enhancing the practicality of psF1. While the sample size required for the normal approximation to be accurate depends on the underlying sensitivity and precision, psF1 automatically determines whether to use the normal approximation based on the number of mass points in the $f_{1}$ distribution to facilitate implementation (the default and recommended option). Simulation studies demonstrated that results obtained using this automatic selection procedure are very close to those based on the exact distribution (Tables S1 and S2). Additionally, psF1 provides options to force the normal approximation (normal.approx = TRUE) or to use the exact distribution (normal.approx = FALSE). Warnings will be issued if users force the normal approximation when it is likely to be unreliable.

Simulation studies and real‐world applications show that psF1 outperforms existing approaches, especially in scenarios with low precision and high sensitivity. These advantages are reflected in shorter interval lengths and higher statistical power in hypothesis testing. Moreover, our simulations confirm that the analytically calculated powers closely align with empirical powers. To further enhance its flexibility, psF1 offers the first extension from the $F_{1}$ score to the more general $F_{\beta }$ score, allowing adjustment of the relative weight between precision and sensitivity. Most importantly, psF1 is the first framework to enable power and sample size calculation for both single and comparative $F_{1}$ and $F_{\beta }$ scores, for which established methods are currently limited. When comparing the $F_{\beta }$ scores of two classifiers, psF1 assumes that both classifiers are evaluated on a shared dataset. It can also accommodate comparisons using two independent datasets, provided they are sufficiently similar. In such cases, a 1:1 allocation ratio would require doubling the sample size. Currently, psF1 is designed for the $F_{\beta }$ score in binary classification tasks. In the future, we plan to extend psF1 to support evaluation metrics for multi‐class classification, such as micro‐, macro‐, and weighted‐$F_{\beta }$ scores.

psF1 can be applied even under extreme class imbalance (Tables S4 and S8). However, caution is required in scenarios with very small number of positives. For interval estimation and hypothesis testing, psF1 remains applicable, but a very small number of positives may lead to wide confidence intervals and non‐significant test results. Moreover, for study design, a very small number of positives may lead to situations in which no $p_{\alpha }$ can achieve a target $F_{1}$ score given the specified precision and sensitivity. In such cases, it may be necessary to increase the number of positives or to adjust the specified precision and sensitivity.

In this study, we assume that predictions for individual instances are independent, which implies independence between true positives and false positives. This assumption may be violated when instances share common backgrounds or environments, such as when multiple instances are from the same subject. In addition, when two classifiers are evaluated on the same dataset, their predictions may be correlated, particularly if the classifiers rely on similar mechanisms, such as when one classifier is an improved version of the other. In such cases, the independence assumption between the predictions of the two classifiers may also be violated. Applying psF1 in settings where instances are correlated or where predictions from two classifiers are correlated may therefore lead to inaccurate confidence intervals and reduced statistical power.

## Funding

This work was supported by National Institutes of Health (P30 CA068485, U2C CA233291, R01 CA252964, U54 CA260560, and CA163072).

## Conflicts of Interest

The authors declare no conflicts of interest.

## Supporting information


**Table S1:** Comparison of the automatic approach, the normal approximation, and the exact approach in terms of mean (standard deviation) of the interval length of 95% CI and coverage probability (shown in the second rows), based on 1000 simulation runs for the $F_{1}$ score of a single classifier. See Section 3.1 for the details of the simulation setting.
**Table S2:** Comparison of the automatic approach, the normal approximation, and the exact approach in terms of mean (standard deviation) of the interval length of 95% CI and coverage probability (shown in the second rows), based on 1000 simulation runs for the difference between two classifiers' $F_{1}$ scores. See Section 3.1 for the details of the simulation setting.
**Table S3:** Mean (standard deviation) of the interval length of 95% CI and coverage probability (shown in the second rows) across 1000 simulation runs for the $F_{1}$ score of a single classifier in scenarios with more extreme sensitivity‐precision tradeoffs.
**Table S4:** Mean (standard deviation) of the interval length of 95% CI and coverage probability (shown in the second rows) across 1000 simulation runs for the $F_{1}$ score of a single classifier in scenarios with very small S/N ratios.
**Table S5:** Mean and standard deviation (SD) of the interval length of 95% CI, as well as coverage probability (C.Prob.) across 1000 simulation runs for the difference between two classifiers' $F_{1}$ scores.
**Table S6:** Type I error comparison between methods for the $F_{1}$ score of a single classifier at the significance level of 0.05, when the null hypothesis is true ($p_{p1}$ = $p_{p0}$, $p_{s1}$ = $p_{s0}$).
**Table S7:** Type I errors comparison between methods for the difference between two classifiers' $F_{1}$ scores at the significance level of 0.05, when the null hypothesis is true.
**Table S8:** Power comparison between methods for the difference between two classifiers' $F_{1}$ scores at the significance level of 0.05 in scenarios with very small S/N ratios.
**Table S9:** Confusion matrices for standard screening and cfDNA testing in the diagnosis of trisomy 21.
**Table S10:** Confusion matrices for three models in the prediction of postoperative urinary retention.

## Data Availability

The data that supports the findings of this study are available in the Supporting Information of this article. The freely available R package psF1 at https://github.com/cyhsuTN/psF1.
