# Supplementary material for: A Unified Framework for Statistical Inference and Power Analysis of Single and Comparative Fβ Scores
Source: Stat Med. 2026 Apr 28;45:e70557. doi: 10.1002/sim.70557 (PMC13125741; doi:10.1002/sim.70557)
Supplement: Supplementary file 1 — Table S1: Comparison of the automatic approach, the normal approximation, and the exact approach in terms of mean (standard deviation) of the interval length of 95% CI and coverage probability (shown in the second rows), based on 1000 simulation runs for the F1 score of a single classifier. See Section 3.1 for the details of the simulation setting. Table S2: Comparison of the automatic approach, the normal approximation, and the exact approach in terms of mean (standard deviation) of the interval length of 95% CI and coverage probability (shown in the second rows), based on 1000 simulation runs for the difference between two classifiers' F1 scores. See Section 3.1 for the details of the simulation setting. Table S3: Mean (standard deviation) of the interval length of 95% CI and coverage probability (shown in the second rows) across 1000 simulation runs for the F1 score of a single classifier in scenarios with more extreme sensitivity‐precision tradeoffs. Table S4: Mean (standard deviation) of the interval length of 95% CI and coverage probability (shown in the second rows) across 1000 simulation runs for the F1 score of a single classifier in scenarios with very small S/N ratios. Table S5: Mean and standard deviation (SD) of the interval length of 95% CI, as well as coverage probability (C.Prob.) across 1000 simulation runs for the difference between two classifiers' F1 scores. Table S6: Type I error comparison between methods for the F1 score of a single classifier at the significance level of 0.05, when the null hypothesis is true (pp1 = pp0, ps1 = ps0). Table S7: Type I errors comparison between methods for the difference between two classifiers' F1 scores at the significance level of 0.05, when the null hypothesis is true. Table S8: Power comparison between methods for the difference between two classifiers' F1 scores at the significance level of 0.05 in scenarios with very small S/N ratios. Table S9: Confusion matrices for standard screening and cfDNA testing i [file SIM-45-0-s001.docx]

**Supplemental Materials**

**A Unified Framework for Statistical Inference and Power Analysis of Single and Comparative** $\boldsymbol{F}_{\boldsymbol{\beta}}$ **Scores**

Chih-Yuan Hsu, Qi Liu*, Yu Shyr*

Department of Biostatistics, Vanderbilt University Medical Center, Nashville, TN 37203, USA

Center for Quantitative Sciences, Vanderbilt University Medical Center, Nashville, TN 37203, USA

*Correspondence: Qi Liu, qi.liu@vumc.org; Yu Shyr, yu.shyr@vumc.org

**METHODS**

**One-sided hypothesis tests**

**For the** $\boldsymbol{F}_{\boldsymbol{1}}$ **score of a single classifier**

In Section 2.1.2.1, when $C\left( u \right)=\sum_{f\leq u} g\left( f;p_{s0}, p_{p0} \right)$ or $=\sum_{f\geq u} g\left( f;p_{s0}, p_{p0} \right)$, it corresponds to a one-sided hypothesis test $H_{0}: F_{1}\geq F_{1}(p_{s0},p_{p0})$ vs $H_{1}: F_{1}<F_{1}(p_{s0},p_{p0})$ or $H_{0}: F_{1}\leq F_{1}(p_{s0},p_{p0})$ vs $H_{1}:F_{1}>F_{1}(p_{s0},p_{p0})$. In Section 2.1.2.2, similarly, $C\left( u \right)=\sum_{f\leq u} g\left( f;p_{s}, p_{p} \right)$ or $=\sum_{f\geq u} g\left( f;p_{s}, p_{p} \right)$ in $p_{v}\left( f_{obs};p_{s}, p_{p} \right)$ corresponds to a one-sided hypothesis test $H_{0}: F_{1}\geq F_{1,0}$ vs $H_{1}: F_{1}<F_{1,0}$ or $H_{0}: F_{1}\leq F_{1,0}$ vs $H_{1}: F_{1}>F_{1,0}$.

**For comparing** $\boldsymbol{F}_{\boldsymbol{1}}$ **scores between two classifiers**

In Sections 2.2.2.1 and 2.2.2.2, When $C_{2}\left( u \right)=\sum_{\Delta f\leq u} h\left( \Delta f;\bar{p}_{s}, \bar{p}_{p}, \bar{p}_{s}, \bar{p}_{p} \right)$ or $=\sum_{\Delta f\geq u} h\left( \Delta f;\bar{p}_{s}, \bar{p}_{p}, \bar{p}_{s}, \bar{p}_{p} \right)$, it corresponds to a one-sided test $H_{0}: F_{1,1}\geq F_{1,2}$ or $H_{0}: F_{1,1}\leq F_{1,2}$.

**Power and sample size calculation for** $\boldsymbol{F}_{\boldsymbol{1}}$ **scores without specified sensitivities and precisions**

**For the** $\boldsymbol{F}_{\boldsymbol{1}}$ **score of a single classifier**

In Section 2.1.3, if the target $F_{1}$ score of $F_{1,0}$ is specified but not limited to be defined by $p_{s0}$ and $p_{p0}$, then the power to detect whether the classifier’s $F_{1}$ score differs from the specified $F_{1,0}$ is given by

$$\sum_{f\in\left\{ f: p_{v}\left( f;F_{1,0} \right)<\alpha^{*} \right\}} g\left( f;p_{s1}, p_{p1} \right). (A1)$$

Furthermore, if the classifier is expected to have an $F_{1}$ score of $F_{1,1}$ without being limited to be defined by $p_{s1}$ and $p_{p1}$, then the power to detect a difference from the specified $F_{1,0}$ is given by

$$\inf_{\left( p_{s1}, p_{p1} \right)\in\Theta\left( F_{1,1} \right)}\sum_{f\in\left\{ f: p_{v}\left( f;F_{1,0} \right)<\alpha^{*} \right\}} g\left( f;p_{s1}, p_{p1} \right). (A2)$$

Under the same $N$, $S$, and $\alpha^{*}$, the power obtained from Formula (6) in the main article is larger than those obtained from Formulas (A1) and (A2) since $p_{v}\left( f;F_{1,0} \right)\geq p_{v}\left( f;p_{s}, p_{p} \right)$ when $F_{1}\left( p_{s0},p_{p0} \right)=F_{1,0}$, and the power given by Formula (A2) is the lowest.

**For comparing** $\boldsymbol{F}_{\boldsymbol{1}}$ **scores between two classifiers**

In Section 2.2.3, if sensitivities and precisions for two classifiers are not specified, the power to detect the difference in $F_{1}$ scores between classifiers 1 and 2 is given by

$$\inf_{\left( p_{s1}, p_{p1},p_{s2}, p_{p2} \right)\in\Theta_{2}\left( F_{1,1},F_{1,2} \right)}\sum_{\Delta f\in\left\{ \Delta f: p_{v2}\left( \Delta f;\bar{p}_{s}, \bar{p}_{p} \right)<\alpha^{*} \right\}} h\left( \Delta f;p_{s1}, p_{p1},p_{s2}, p_{p2} \right), (A3)$$

where $\Theta_{2}\left( F_{1,1},F_{1,2} \right)=\left\{ \left( p_{s1}, p_{p1},p_{s2}, p_{p2} \right):\left( p_{s1}, p_{p1} \right)\in\Theta\left( F_{1,1} \right), \left( p_{s2}, p_{p2} \right)\in\Theta\left( F_{1,2} \right) \right\}$.

**REAL APPLICATION**

*Application 2: Prediction of postoperative urinary retention (POUR)*

POUR is a common complication following spine surgery and is associated with increased rates of urinary tract infection, bladder overdistention, and autonomic dysregulation. To predict the occurrence of POUR post spine surgery, Porche et al. (2022) proposed three models using data collected from 1,311 adult patients who underwent lumbar spine surgery at the University of Florida between June 1, 2017, and June 1, 2019. After excluding patients who did not meet the inclusion criteria, 891 patients were retained for model development. The dataset was divided into training, validation, and testing sets in a 65:10:25 ratio. The three models were based on a binomial logistic regression (Model 1), an artificial neural network (Model 2), and a hybrid model combining both methods (Model 3). Model performance was evaluated using a test set of 176 patients without POUR and 59 patients with POUR. The hybrid model showed the best performance, with an area under the curve (AUC) of 0.753, compared to 0.737 for the logistic regression model and 0.735 for the neural network model.

Besides, using the optimal probability cutoffs provided in their paper, Model 1 achieved a specificity of 85.2%, sensitivity of 49.2%, and PPV of 52.7%, with 29 TPs and 26 FPs. Model 2 yielded a specificity of 54.5%, sensitivity of 84.7%, and PPV of 38.5%, with 50 TPs and 108 FPs. Model 3 reached a specificity of 68.2%, sensitivity of 72.9%, and PPV of 43.4%, resulting from 43 TPs and 56 FPs (Supplemental Table S10). Based on these results, we evaluated the three models in terms of $F_{1}$ scores. The estimated $F_{1}$ scores for the three models were 0.509, 0.461, and 0.544, respectively. Notably, the ranking of the models by $F_{1}$ score aligns with their ranking by AUC values; however, the differences in $F_{1}$ scores are more pronounced. Interestingly, the estimated accuracies for the models were 0.761, 0.502, and 0.694, respectively, with Model 1 achieving the highest accuracy. This superior accuracy is primarily due to Model 1’s higher specificity, which has a greater influence on accuracy in the imbalanced test set containing more non-POUR than POUR patients.

Next, we tested whether the $F_{1}$ score of Model 3 was significantly higher than those of Models 1 and 2 using psF1. Based on one-sided tests, the p-values for comparing Model 3 against Model 1 and Model 2 were 0.295 and 0.031, respectively, while the p-value for comparing Model 1 against Model 2 is 0.204. In contrast, using the Takahashi approach, the p-values for Model 3 against Model 1 and Model 2 are 0.317 and 0.095, respectively, and 0.250 for Model 1 vs Model 2. These results indicate that, according to psF1, Model 3 performs significantly better than Model 2 at the 5% significance level. While Model 3 also shows improved performance over Model 1, the difference is not statistically significant.

Given the observed sensitivity and PPV values, redesigning the study to detect the difference in $F_{1}$ scores between Model 3 and Model 1 with 80% power at a 5% one-sided significance level would require both the sample size $N$ and the number of actual positives $S$ to be 22 times larger than those used in the current analysis. In contrast, if the focus shifts toward sensitivity, such as evaluating the difference in $F_{2}$ scores, the required $N$ and $S$ would increase by only about 1.8 times.

In addition, we performed statistical inference on the $F_{1}$ score of the best-performing Model 3 using the psF1-CI and compare it with existing CI methods in terms of interval length. The 95% CI estimated by psF1-CI was (0.467, 0.618), while the corresponding 95% CIs estimated by CP, Wald, WD, and WID were (0.444, 0.639), (0.451, 0.638), (0.444, 0.630), and (0.451, 0.635), respectively. The interval lengths for psF1-CI, CP, Wald, WD, and WID were 0.151, 0.194, 0.187, 0.185, and 0.184, respectively. Notably, psF1-CI yielded the shortest interval, indicating greater precision compared to the alternative methods. For reference, the 95% CrI estimated by the Wang’s Bayesian approach was (0.448, 0.632), with a length of 0.184.

**ARTIFICIAL EXAMPLES**

We will guide users on how to use the proposed methods with R commands.

**Methods for the** $\boldsymbol{F}_{\boldsymbol{1}}$ **score of a single classifier**

***Confidence interval***

Suppose we observe the confusion matrix of the new classifier as follows:

|  |  | Predicted | |  |
| --- | --- | --- | --- | --- |
|  |  | **Negative** | **Positive** |  |
| **Actual** | **Negative** | 45 | 1 | 46 |
|  | **Positive** | 2 | 10 | 12 |
|  |  | 47 | 11 | 58 |

The 95% confidence interval for the $F_{1}$ score of the new classifier is given by (0.649, 1.000), and the point estimate for the $F_{1}$ score is 0.840 (calculated by $\tilde{p}_{s}$ and $\tilde{p}_{p}$ under Jeffrey’s non-informative prior). The point estimate of 0.870 is calculated using $\hat{p}_{s}=d_{obs}/S$ and $\hat{p}_{p}=d_{obs}/(b_{obs}+d_{obs})$.

ciFbOne(d=10, b=1, N=58, s=12,

beta=1,

alpha=0.05)

## Fb.est 95%CI.L 95%CI.U Fb.est.raw

## 0.8400000 0.6486486 1.0000000 0.8695652

***Hypothesis testing***

Based on the observed confusion matrix, we evaluate whether the $F_{1}$ score of the new classifier significantly differs from the target $F_{1}$ score defined by $p_{p}$=9/11 and $p_{s}$=0.75. The testing result shows that the difference is not significant at the 5% two-sided significance level (p-value = 0.427).

testFbOne(d=10, b=1, N=58, s=12,

Fb=NULL, pp=9/11, ps=0.75,

beta=1,

alternative=c("two.sided", "less", "greater")[1])

## fb P-value Fb pp ps

## 0.86957 0.42744 0.78261 0.81818 0.75000

What if the question is to test whether the $F_{1}$ score of the new classifier significantly differs from the target $F_{1}$ score of 0.783? The test shows a non-significant result at the 5% two-sided significance level (p-value = 0.434).

testFbOne(d=10, b=1, N=58, s=12,

Fb=0.783, pp=NULL, ps=NULL,

beta=1,

alternative=c("two.sided", "less", "greater")[1])

## fb P-value Fb pp.argsup ps.argsup

## 0.86957 0.43398 0.78300 0.83000 0.74104

***Power and sample size calculation***

At a 5% two-sided significance level, how many classification instances ($N$) and positives ($S$) are required to achieve 80% statistical power to detect a significant difference between a new classifier’s $F_{1}$ score, defined by $p_{p1}$=0.9 and $p_{s1}$=0.85, and the target $F_{1}$ score, defined by $p_{p}$=9/11 and ps=0.75? The answer is $N$ = 377 and $S$ = 78.

powerFbOne(N=round(58*6.5), s=round(12*6.5),

Fb1=NULL, pp1=0.90, ps1=0.85,

Fb=NULL, pp=9/11, ps=0.75,

beta=1,

alternative=c("two.sided", "less", "greater")[1],

alpha.level=0.05)

## Power pp ps Fb pp1 ps1 Fb1

## 0.80560 0.81818 0.75000 0.78261 0.90000 0.85000 0.87429

At a 5% two-sided significance level, how many classification instances ($N$) and positives ($S$) are required to achieve 80% statistical power to detect a significant difference between a new classifier’s $F_{1}$ score of 0.874 and the target $F_{1}$ score defined by $p_{p}$=9/11 and $p_{s}$=0.75? The answer is $N$ = 394 and $S$ = 82.

powerFbOne(N=round(58*6.8), s=round(12*6.8),

Fb1=0.874, pp1=NULL, ps1=NULL,

Fb=NULL, pp=9/11, ps=0.75,

beta=1,

alternative=c("two.sided", "less", "greater")[1],

alpha.level=0.05)

## Power pp ps Fb pp1 ps1 Fb1

## 0.80005 0.81818 0.75000 0.78261 1.00000 0.77620 0.87400

At a 5% two-sided significance level, how many classification instances ($N$) and positives ($S$) are required to achieve 80% statistical power to detect a significant difference between a new classifier’s $F_{1}$ score of 0.874 and the target $F_{1}$ score of 0.783? The answer is $N$ = 476 and $S$ = 98.

powerFbOne(N=round(58*8.2), s=round(12*8.2),

Fb1=0.874, pp1=NULL, ps1=NULL,

Fb=0.783, pp=NULL, ps=NULL,

beta=1,

alternative=c("two.sided", "less", "greater")[1],

alpha.level=0.05)

## Power pp ps Fb pp1 ps1 Fb1

## 0.80799 0.95000 0.66594 0.78300 1.00000 0.77620 0.87400

**Methods for the comparison in** $\boldsymbol{F}_{\boldsymbol{1}}$ **scores between two classifiers**

***Confidence interval***

Suppose we observe the confusion matrices of the two classifiers:

|  |  | Classifier 1 | |  |  | Classifier 2 | |  |
| --- | --- | --- | --- | --- | --- | --- | --- | --- |
|  |  | **Negative** | **Positive** |  |  | **Negative** | **Positive** |  |
| **Actual** | **Negative** | 45 | 1 |  |  | 44 | 2 | 46 |
|  | **Positive** | 2 | 10 |  |  | 3 | 9 | 12 |
|  |  | 47 | 11 |  |  | 47 | 11 | 58 |

The 95% confidence interval for the $F_{1}$ score difference is given by (-0.169, 0.333), and the point estimate for the $F_{1}$ score difference is 0.080 (calculated by $\tilde{p}_{sk}$ and $\tilde{p}_{pk}$ under Jeffrey’s non-informative prior). The point estimate of 0.087 is calculated using $\hat{p}_{sk}=d_{obs,k}/S$ and $\hat{p}_{pk}=d_{obs,k}/(b_{obs,k}+d_{obs,k})$.

ciFbTwo(d1=10, b1=1,

d2=9, b2=2,

N=58, s=12,

beta=1,

alpha=0.05)

## dFb.est 95%CI.L 95%CI.U dFb.est.raw

## 0.08000000 -0.16879795 0.33333333 0.08695652

***Hypothesis testing***

Based on the observed confusion matrices, we evaluate whether the $F_{1}$ score of Classifier 1 significantly differs from the $F_{1}$ score of Classifier 2. When generating the null distribution of $\Delta f_{1}$ using $F_{1,1}=F_{1,2}=\hat{F}_{1,0}$, the test shows that the difference is not significant at the 5% two-sided significance level (p-value = 0.478).

testFbTwo(d1=10, b1=1,

d2=9, b2=2,

Fb="est",

N=58, s=12,

beta=1,

alternative=c("two.sided", "less", "greater")[1])

## fb1 fb2 dfb P-value Fb0.est pp.bar ps.bar

## 0.86957 0.78261 0.08696 0.47818 0.82609 0.86364 0.79167

When generating the null distribution of $\Delta f_{1}$ using $F_{1,1}=F_{1,2}=F_{1,0}$ for all possible $F_{1,0}$, the test shows a non-significant result at the 5% two-sided significance level (p-value = 0.677).

testFbTwo(d1=10, b1=1,

d2=9, b2=2,

Fb=NULL,

N=58, s=12,

beta=1,

alternative=c("two.sided", "less", "greater")[1])

## fb1 fb2 dfb P-value Fb0.argsup pp.argsup ps.argsup

## 0.86957 0.78261 0.08696 0.67718 0.60000 1.00000 0.42857

***Power and sample size calculation***

At a 5% two-sided significance level, how many classification instances ($N$) and positives ($S$) are required to achieve 80% statistical power to detect a significant difference between Classifier 1’s $F_{1}$ score, defined by $p_{p1}$=0.9 and $p_{s1}$=0.85, and Classifier 2’s $F_{1}$ score, defined by $p_{p2}$=9/11 and $p_{s2}$=0.75? The answer is $N$ = 754 and $S$ = 156.

powerFbTwo(N=round(58*13), s=round(12*13),

Fb1=NULL, pp1=0.90, ps1=0.85,

Fb2=NULL, pp2=9/11, ps2=0.75,

beta=1,

alternative=c("two.sided", "less", "greater")[1],

alpha.level=0.05)

## Power pp1 ps1 Fb1 pp2 ps2 Fb2

## 0.80537 0.90000 0.85000 0.87429 0.81818 0.75000 0.78261

**Supplemental Tables**

**Table S1.** Comparison of the automatic approach, the normal approximation, and the exact approach in terms of mean (standard deviation) of the interval length of 95% CI and coverage probability (shown in the second rows), based on 1000 simulation runs for the $F_{1}$ score of a single classifier. See Section 3.1 for the details of the simulation setting.

| $\boldsymbol{p}_{\boldsymbol{p}\boldsymbol{1}}$=0.6, $\boldsymbol{p}_{\boldsymbol{s}\boldsymbol{1}}$=0.9 ($\boldsymbol{F}_{\boldsymbol{1}\boldsymbol{,}\boldsymbol{1}}$=0.720) | | | |
| --- | --- | --- | --- |
|  | **auto** | **TRUE** | **FALSE** |
| N=150, S=60 | 0.120 (0.010)  0.943 | 0.119 (0.010)  0.944 | 0.120 (0.010)  0.943 |
| N=100, S=40 | 0.148 (0.015)  0.951 | 0.147 (0.015)  0.952 | 0.148 (0.015)  0.951 |
| N=50, S=20 | 0.219 (0.029)  0.959 | 0.212 (0.029)  0.959 | 0.219 (0.029)  0.959 |
| N=1000, S=20 | 0.248 (0.023)  0.961 | 0.242 (0.023)  0.955 | 0.248 (0.023)  0.961 |
| $\boldsymbol{p}_{\boldsymbol{p}\boldsymbol{1}}$**=0.9,** $\boldsymbol{p}_{\boldsymbol{s}\boldsymbol{1}}$**=0.6 (**$\boldsymbol{F}_{\boldsymbol{1}\boldsymbol{,}\boldsymbol{1}}$**=0.720)** | | | |
|  | **auto** | **TRUE** | **FALSE** |
| N=150, S=60 | 0.202 (0.018)  0.943 | 0.200 (0.018)  0.947 | 0.202 (0.018)  0.943 |
| N=100, S=40 | 0.248 (0.026)  0.952 | 0.246 (0.027)  0.948 | 0.248 (0.026)  0.952 |
| N=50, S=20 | 0.358 (0.050)  0.962 | 0.352 (0.051)  0.946 | 0.358 (0.050)  0.962 |
| N=1000, S=20 | 0.358 (0.050)  0.963 | 0.352 (0.051)  0.945 | 0.358 (0.050)  0.963 |
| $\boldsymbol{p}_{\boldsymbol{p}\boldsymbol{1}}$**=0.72,** $\boldsymbol{p}_{\boldsymbol{s}\boldsymbol{1}}$**=0.72 (**$\boldsymbol{F}_{\boldsymbol{1}\boldsymbol{,}\boldsymbol{1}}$**=0.720)** | | | |
|  | **auto** | **TRUE** | **FALSE** |
| N=150, S=60 | 0.172 (0.014)  0.954 | 0.171 (0.014)  0.952 | 0.172 (0.014)  0.954 |
| N=100, S=40 | 0.211 (0.021)  0.954 | 0.210 (0.021)  0.952 | 0.211 (0.021)  0.954 |
| N=50, S=20 | 0.306 (0.041)  0.953 | 0.299 (0.042)  0.952 | 0.306 (0.041)  0.953 |
| N=1000, S=20 | 0.313 (0.039)  0.958 | 0.307 (0.040)  0.952 | 0.313 (0.039)  0.958 |

**Table S2.** Comparison of the automatic approach, the normal approximation, and the exact approach in terms of mean (standard deviation) of the interval length of 95% CI and coverage probability (shown in the second rows), based on 1000 simulation runs for the difference between two classifiers’ $F_{1}$ scores. See Section 3.1 for the details of the simulation setting.

| $\boldsymbol{p}_{\boldsymbol{p}\boldsymbol{1}}$=0.5, $\boldsymbol{p}_{\boldsymbol{s}\boldsymbol{1}}$=0.8 ($\boldsymbol{F}_{\boldsymbol{1}\boldsymbol{,}\boldsymbol{1}}$=0.615); $\boldsymbol{p}_{\boldsymbol{p}\boldsymbol{2}}$=0.6, $\boldsymbol{p}_{\boldsymbol{s}\boldsymbol{2}}$=0.9 ($\boldsymbol{F}_{\boldsymbol{1}\boldsymbol{,}\boldsymbol{2}}$=0.720) | | | |
| --- | --- | --- | --- |
|  | **auto** | **TRUE** | **FALSE** |
| N=250, S=100 | 0.137 (0.007)  0.948 | 0.137 (0.007)  0.948 | 0.137 (0.007)  0.948 |
| N=100, S=40 | 0.219 (0.016)  0.958 | 0.219 (0.016)  0.958 | 0.219 (0.016)  0.954 |
| N=50, S=20 | 0.316 (0.032)  0.959 | 0.315 (0.032)  0.961 | 0.316 (0.032)  0.959 |
| N=1000, S=20 | 0.360 (0.024)  0.958 | 0.360 (0.024)  0.958 | 0.360 (0.024)  0.958 |
| $\boldsymbol{p}_{\boldsymbol{p}\boldsymbol{1}}$**=0.5,** $\boldsymbol{p}_{\boldsymbol{s}\boldsymbol{1}}$**=0.9 (**$\boldsymbol{F}_{\boldsymbol{1}\boldsymbol{,}\boldsymbol{1}}$**=0.643);** $\boldsymbol{p}_{\boldsymbol{p}\boldsymbol{2}}$**=0.6,** $\boldsymbol{p}_{\boldsymbol{s}\boldsymbol{2}}$**=0.9 (**$\boldsymbol{F}_{\boldsymbol{1},\boldsymbol{2}}$**=0.720)** | | | |
|  | **auto** | **TRUE** | **FALSE** |
| N=250, S=100 | 0.121 (0.006)  0.953 | 0.121 (0.006)  0.953 | 0.121 (0.006)  0.953 |
| N=100, S=40 | 0.194 (0.015)  0.954 | 0.194 (0.015)  0.954 | 0.194 (0.015)  0.952 |
| N=50, S=20 | 0.283 (0.029)  0.959 | 0.282 (0.029)  0.963 | 0.283 (0.029)  0.959 |
| N=1000, S=20 | 0.337 (0.021)  0.962 | 0.337 (0.021)  0.962 | 0.337 (0.021)  0.962 |
| $\boldsymbol{p}_{\boldsymbol{p}\boldsymbol{1}}$**=0.9,** $\boldsymbol{p}_{\boldsymbol{s}\boldsymbol{1}}$**=0.7 (**$\boldsymbol{F}_{\boldsymbol{1}\boldsymbol{,}\boldsymbol{1}}$**=0.788);** $\boldsymbol{p}_{\boldsymbol{p}\boldsymbol{2}}$**=0.9,** $\boldsymbol{p}_{\boldsymbol{s}\boldsymbol{2}}$**=0.9 (**$\boldsymbol{F}_{\boldsymbol{1},\boldsymbol{2}}$**=0.9)** | | | |
|  | **auto** | **TRUE** | **FALSE** |
| N=250, S=100 | 0.157 (0.010)  0.952 | 0.157 (0.010)  0.952 | 0.157 (0.010)  0.952 |
| N=100, S=40 | 0.252 (0.024)  0.948 | 0.252 (0.024)  0.950 | 0.252 (0.024)  0.948 |
| N=50, S=20 | 0.366 (0.046)  0.965 | 0.365 (0.046)  0.969 | 0.366 (0.046)  0.965 |
| N=1000, S=20 | 0.369 (0.046)  0.966 | 0.368 (0.046)  0.969 | 0.369 (0.046)  0.966 |

**Table S3.** Mean (standard deviation) of the interval length of 95% CI and coverage probability (shown in the second rows) across 1000 simulation runs for the $F_{1}$ score of a single classifier in scenarios with more extreme sensitivity-precision tradeoffs.

| $\boldsymbol{p}_{\boldsymbol{p}\boldsymbol{1}}$=0.4, $\boldsymbol{p}_{\boldsymbol{s}\boldsymbol{1}}$=0.9 ($\boldsymbol{F}_{\boldsymbol{1}\boldsymbol{,}\boldsymbol{1}}$=0.554) | | | | | |
| --- | --- | --- | --- | --- | --- |
|  | **psF1-CI** | **CP** | **Wald** | **WD** | **WID** |
| N=150, S=60 | 0.077 (0.011)  0.948 | 0.174 (0.003)  1.000 | 0.168 (0.003)  1.000 | 0.166 (0.002)  1.000 | 0.165 (0.002)  1.000 |
| N=100, S=40 | 0.097 (0.017)  0.947 | 0.214 (0.004)  1.000 | 0.205 (0.004)  1.000 | 0.203 (0.003)  1.000 | 0.201 (0.004)  1.000 |
| N=50, S=20 | 0.146 (0.032)  0.970 | 0.304 (0.008)  1.000 | 0.290 (0.008)  1.000 | 0.283 (0.006)  1.000 | 0.279 (0.007)  1.000 |
| N=1000, S=20 | 0.218 (0.021)  0.966 | 0.302 (0.010)  0.997 | 0.289 (0.009)  0.989 | 0.282 (0.009)  0.990 | 0.277 (0.008)  0.991 |
| $\boldsymbol{p}_{\boldsymbol{p}\boldsymbol{1}}$**=0.9,** $\boldsymbol{p}_{\boldsymbol{s}\boldsymbol{1}}$**=0.4 (**$\boldsymbol{F}_{\boldsymbol{1}\boldsymbol{,}\boldsymbol{1}}$**=0.554)** | | | | | |
|  | **psF1-CI** | **CP** | **Wald** | **WD** | **WID** |
| N=150, S=60 | 0.253 (0.014)  0.959 | 0.262 (0.014)  0.963 | 0.251 (0.014)  0.949 | 0.246 (0.011)  0.946 | 0.243 (0.013)  0.954 |
| N=100, S=40 | 0.313 (0.023)  0.955 | 0.321 (0.021)  0.971 | 0.306 (0.021)  0.929 | 0.297 (0.015)  0.953 | 0.293 (0.019)  0.951 |
| N=50, S=20 | 0.438 (0.042)  0.963 | 0.450 (0.038)  0.967 | 0.430 (0.042)  0.932 | 0.403 (0.023)  0.949 | 0.394 (0.330)  0.944 |
| N=1000, S=20 | 0.438 (0.042)  0.963 | 0.450 (0.038)  0.967 | 0.430 (0.042)  0.932 | 0.403 (0.023)  0.949 | 0.394 (0.330)  0.944 |
| $\boldsymbol{p}_{\boldsymbol{p}\boldsymbol{1}}$**=0.554,** $\boldsymbol{p}_{\boldsymbol{s}\boldsymbol{1}}$**=0.554 (**$\boldsymbol{F}_{\boldsymbol{1}\boldsymbol{,}\boldsymbol{1}}$**=0.554)** | | | | | |
|  | **psF1-CI** | **CP** | **Wald** | **WD** | **WID** |
| N=150, S=60 | 0.199 (0.012)  0.951 | 0.222 (0.010)  0.974 | 0.213 (0.009)  0.963 | 0.210 (0.008)  0.964 | 0.208 (0.009)  0.968 |
| N=100, S=40 | 0.245 (0.018)  0.953 | 0.272 (0.014)  0.974 | 0.260 (0.014)  0.964 | 0.255 (0.011)  0.965 | 0.252 (0.013)  0.968 |
| N=50, S=20 | 0.350 (0.036)  0.956 | 0.384 (0.027)  0.977 | 0.366 (0.028)  0.945 | 0.351 (0.019)  0.967 | 0.343 (0.023)  0.970 |
| N=1000, S=20 | 0.359 (0.033)  0.952 | 0.384 (0.027)  0.973 | 0.365 (0.029)  0.936 | 0.350 (0.020)  0.963 | 0.343 (0.024)  0.962 |

**Table S4.** Mean (standard deviation) of the interval length of 95% CI and coverage probability (shown in the second rows) across 1000 simulation runs for the $F_{1}$ score of a single classifier in scenarios with very small $S/N$ ratios.

| $\boldsymbol{p}_{\boldsymbol{p}\boldsymbol{1}}$=0.6, $\boldsymbol{p}_{\boldsymbol{s}\boldsymbol{1}}$=0.9 ($\boldsymbol{F}_{\boldsymbol{1}\boldsymbol{,}\boldsymbol{1}}$=0.720) | | | | | |
| --- | --- | --- | --- | --- | --- |
|  | **psF1-CI** | **CP** | **Wald** | **WD** | **WID** |
| N=8000, S=80  S/N=1% | 0.120 (0.006)  0.953 | 0.146 (0.005)  0.988 | 0.141 (0.005)  0.977 | 0.141 (0.005)  0.986 | 0.139 (0.005)  0.984 |
| N=8000, S=60  S/N=0.75% | 0.139 (0.008)  0.956 | 0.169 (0.007)  0.984 | 0.162 (0.007)  0.974 | 0.163 (0.007)  0.978 | 0.160 (0.007)  0.981 |
| N=8000, S=40  S/N=0.5% | 0.171 (0.012)  0.950 | 0.209 (0.011)  0.979 | 0.198 (0.011)  0.971 | 0.200 (0.010)  0.973 | 0.195 (0.010)  0.973 |
| N=8000, S=20  S/N=0.25% | 0.249 (0.022)  0.960 | 0.298 (0.021)  0.985 | 0.279 (0.023)  0.970 | 0.284 (0.017)  0.970 | 0.269 (0.019)  0.974 |
| $\boldsymbol{p}_{\boldsymbol{p}\boldsymbol{1}}$**=0.9,** $\boldsymbol{p}_{\boldsymbol{s}\boldsymbol{1}}$**=0.6 (**$\boldsymbol{F}_{\boldsymbol{1}\boldsymbol{,}\boldsymbol{1}}$**=0.720)** | | | | | |
|  | **psF1-CI** | **CP** | **Wald** | **WD** | **WID** |
| N=8000, S=80  S/N=1% | 0.173 (0.014)  0.953 | 0.180 (0.014)  0.958 | 0.172 (0.014)  0.940 | 0.173 (0.013)  0.951 | 0.169 (0.013)  0.951 |
| N=8000, S=60  S/N=0.75% | 0.202 (0.018)  0.943 | 0.210 (0.019)  0.956 | 0.199 (0.018)  0.940 | 0.200 (0.017)  0.945 | 0.195 (0.017)  0.943 |
| N=8000, S=40  S/N=0.5% | 0.249 (0.027)  0.952 | 0.259 (0.028)  0.965 | 0.243 (0.028)  0.940 | 0.246 (0.024)  0.951 | 0.237 (0.025)  0.948 |
| N=8000, S=20  S/N=0.25% | 0.360 (0.052)  0.963 | 0.368 (0.053)  0.959 | 0.341 (0.057)  0.921 | 0.347 (0.041)  0.956 | 0.323 (0.046)  0.953 |
| $\boldsymbol{p}_{\boldsymbol{p}\boldsymbol{1}}$=0.72, $\boldsymbol{p}_{\boldsymbol{s}\boldsymbol{1}}$=0.72 ($\boldsymbol{F}_{\boldsymbol{1}\boldsymbol{,}\boldsymbol{1}}$=0.720) | | | | | |
|  | **psF1-CI** | **CP** | **Wald** | **WD** | **WID** |
| N=8000, S=80  S/N=1% | 0.152 (0.010)  0.949 | 0.164 (0.010)  0.964 | 0.157 (0.010)  0.949 | 0.158 (0.009)  0.956 | 0.155 (0.010)  0.955 |
| N=8000, S=60  S/N=0.75% | 0.176 (0.014)  0.957 | 0.191 (0.013)  0.968 | 0.181 (0.013)  0.946 | 0.183 (0.012)  0.958 | 0.179 (0.013)  0.961 |
| N=8000, S=40  S/N=0.5% | 0.217 (0.020)  0.949 | 0.235 (0.020)  0.967 | 0.222 (0.020)  0.941 | 0.224 (0.018)  0.964 | 0.217 (0.019)  0.958 |
| N=8000, S=20  S/N=0.25% | 0.312 (0.039)  0.957 | 0.335 (0.039)  0.968 | 0.311 (0.041)  0.934 | 0.317 (0.031)  0.954 | 0.298 (0.035)  0.956 |

**Table S5.** Mean and standard deviation (SD) of the interval length of 95% CI, as well as coverage probability (C.Prob.) across 1000 simulation runs for the difference between two classifiers’ $F_{1}$ scores.

| $\boldsymbol{p}_{\boldsymbol{p}\boldsymbol{1}}$=0.5, $\boldsymbol{p}_{\boldsymbol{s}\boldsymbol{1}}$=0.8 ($\boldsymbol{F}_{\boldsymbol{1}\boldsymbol{,}\boldsymbol{1}}$=0.615); $\boldsymbol{p}_{\boldsymbol{p}\boldsymbol{2}}$=0.6, $\boldsymbol{p}_{\boldsymbol{s}\boldsymbol{2}}$=0.9 ($\boldsymbol{F}_{\boldsymbol{1}\boldsymbol{,}\boldsymbol{2}}$=0.720) | | | |
| --- | --- | --- | --- |
|  | **Mean** | **SD** | **C.Prob.** |
| N=250, S=100 | 0.137 | 0.007 | 0.948 |
| N=100, S=40 | 0.219 | 0.016 | 0.958 |
| N=50, S=20 | 0.316 | 0.032 | 0.959 |
| N=1000, S=20 | 0.360 | 0.024 | 0.958 |
| $\boldsymbol{p}_{\boldsymbol{p}\boldsymbol{1}}$**=0.5,** $\boldsymbol{p}_{\boldsymbol{s}\boldsymbol{1}}$**=0.9 (**$\boldsymbol{F}_{\boldsymbol{1}\boldsymbol{,}\boldsymbol{1}}$**=0.643);** $\boldsymbol{p}_{\boldsymbol{p}\boldsymbol{2}}$**=0.6,** $\boldsymbol{p}_{\boldsymbol{s}\boldsymbol{2}}$**=0.9 (**$\boldsymbol{F}_{\boldsymbol{1},\boldsymbol{2}}$**=0.720)** | | | |
|  | **Mean** | **SD** | **C.Prob.** |
| N=250, S=100 | 0.121 | 0.006 | 0.953 |
| N=100, S=40 | 0.194 | 0.015 | 0.954 |
| N=50, S=20 | 0.283 | 0.029 | 0.959 |
| N=1000, S=20 | 0.337 | 0.021 | 0.962 |
| $\boldsymbol{p}_{\boldsymbol{p}\boldsymbol{1}}$**=0.9,** $\boldsymbol{p}_{\boldsymbol{s}\boldsymbol{1}}$**=0.7 (**$\boldsymbol{F}_{\boldsymbol{1}\boldsymbol{,}\boldsymbol{1}}$**=0.788);** $\boldsymbol{p}_{\boldsymbol{p}\boldsymbol{2}}$**=0.9,** $\boldsymbol{p}_{\boldsymbol{s}\boldsymbol{2}}$**=0.9 (**$\boldsymbol{F}_{\boldsymbol{1},\boldsymbol{2}}$**=0.9)** | | | |
|  | **Mean** | **SD** | **C.Prob.** |
| N=250, S=100 | 0.157 | 0.010 | 0.952 |
| N=100, S=40 | 0.252 | 0.024 | 0.948 |
| N=50, S=20 | 0.366 | 0.046 | 0.965 |
| N=1000, S=20 | 0.369 | 0.046 | 0.966 |

**Table S6.** Type I error comparison between methods for the $F_{1}$ score of a single classifier at the significance level of 0.05, when the null hypothesis is true ($p_{p1}$=$p_{p0}$, $p_{s1}$=$p_{s0}$).

| $\boldsymbol{p}_{\boldsymbol{p}\boldsymbol{0}}$=0.5, $\boldsymbol{p}_{\boldsymbol{s}\boldsymbol{0}}$=0.8 ($\boldsymbol{F}_{\boldsymbol{1}\boldsymbol{,}\boldsymbol{0}}$=0.615) | | | | | | | | | | | | | | | | | | | | |
| --- | --- | --- | --- | --- | --- | --- | --- | --- | --- | --- | --- | --- | --- | --- | --- | --- | --- | --- | --- | --- |
|  | | **psF1** | | **psF1-CI** | | | **CP** | | | **Wald** | | | **WD** | | | | | **WID** | | **Wang*** |
| N=150, S=60 | | 0.050 | | 0.042 | | | 0.004 | | | 0.006 | | | 0.006 | | | | | 0.005 | | 0.006 |
| N=100, S=40 | | 0.043 | | 0.048 | | | 0.006 | | | 0.008 | | | 0.011 | | | | | 0.009 | | 0.011 |
| N=50, S=20 | | 0.048 | | 0.034 | | | 0.006 | | | 0.013 | | | 0.012 | | | | | 0.010 | | 0.011 |
| N=1000, S=20 | | 0.046 | | 0.040 | | | 0.016 | | | 0.032 | | | 0.024 | | | | | 0.026 | | 0.022 |
| $\boldsymbol{p}_{\boldsymbol{p}\boldsymbol{0}}$**=0.5,** $\boldsymbol{p}_{\boldsymbol{s}\boldsymbol{0}}$**=0.9 (**$\boldsymbol{F}_{\boldsymbol{1}\boldsymbol{,}\boldsymbol{0}}$**=0.643)** | | | | | | | | | | | | | | | | | | | | |
|  | **psF1** | | | | **psF1-CI** | | | **CP** | | | **Wald** | | | **WD** | | **WID** | | | **Wang*** | |
| N=150, S=60 | 0.053 | | | | 0.041 | | | 0.000 | | | 0.001 | | | 0.001 | | 0.001 | | | 0.000 | |
| N=100, S=40 | 0.048 | | | | 0.039 | | | 0.000 | | | 0.001 | | | 0.004 | | 0.002 | | | 0.003 | |
| N=50, S=20 | 0.052 | | | | 0.032 | | | 0.000 | | | 0.002 | | | 0.003 | | 0.000 | | | 0.001 | |
| N=1000, S=20 | 0.048 | | | | 0.046 | | | 0.012 | | | 0.021 | | | 0.021 | | 0.014 | | | 0.018 | |
| $\boldsymbol{p}_{\boldsymbol{p}\boldsymbol{0}}$**=0.9,** $\boldsymbol{p}_{\boldsymbol{s}\boldsymbol{0}}$**=0.7 (**$\boldsymbol{F}_{\boldsymbol{1}\boldsymbol{,}\boldsymbol{0}}$**=0.788)** | | | | | | | | | | | | | | | | | | | | |
|  | | | **psF1** | | | **psF1-CI** | | | **CP** | | | **Wald** | | | **WD** | | **WID** | | **Wang*** | |
| N=150, S=60 | | | 0.046 | | | 0.050 | | | 0.040 | | | 0.062 | | | 0.048 | | 0.046 | | 0.049 | |
| N=100, S=40 | | | 0.050 | | | 0.047 | | | 0.038 | | | 0.066 | | | 0.056 | | 0.046 | | 0.051 | |
| N=50, S=20 | | | 0.049 | | | 0.044 | | | 0.029 | | | 0.079 | | | 0.054 | | 0.049 | | 0.054 | |
| N=1000, S=20 | | | 0.049 | | | 0.044 | | | 0.029 | | | 0.080 | | | 0.054 | | 0.049 | | 0.054 | |

*Power: the probability that CrI does not include the null $F_{1}$ value.

**Table S7.** Type I errors comparison between methods for the difference between two classifiers’ $F_{1}$ scores at the significance level of 0.05, when the null hypothesis is true.

| $\boldsymbol{p}_{\boldsymbol{p}\boldsymbol{1}}$=0.5, $\boldsymbol{p}_{\boldsymbol{s}\boldsymbol{1}}$=0.8 ($\boldsymbol{F}_{\boldsymbol{1}\boldsymbol{,}\boldsymbol{1}}$=0.615) | | | | | | | | | |
| --- | --- | --- | --- | --- | --- | --- | --- | --- | --- |
|  | **psF1** | **psF1-CI** | | **Permutation** | | | **G&G*** | | **Takahashi** |
| $\boldsymbol{p}_{\boldsymbol{p}\boldsymbol{2}}$**=**$\boldsymbol{p}_{\boldsymbol{p}\boldsymbol{1}}$**,** $\boldsymbol{p}_{\boldsymbol{s}\boldsymbol{2}}$**=**$\boldsymbol{p}_{\boldsymbol{s}\boldsymbol{1}}$ |  |  | |  | | |  | |  |
| N=250, S=100 | 0.045 | 0.042 | | 0.048 | | | 0.005 | | 0.007 |
| N=100, S=40 | 0.038 | 0.031 | | 0.052 | | | 0.005 | | 0.005 |
| N=50, S=20 | 0.039 | 0.025 | | 0.042 | | | 0.004 | | 0.005 |
| N=1000, S=20 | 0.037 | 0.027 | | 0.044 | | | 0.006 | | 0.012 |
| $\boldsymbol{p}_{\boldsymbol{p}\boldsymbol{2}}$=$\boldsymbol{p}_{\boldsymbol{s}\boldsymbol{1}}$, $\boldsymbol{p}_{\boldsymbol{s}\boldsymbol{2}}$=$\boldsymbol{p}_{\boldsymbol{p}\boldsymbol{1}}$ |  |  | |  | | |  | |  |
| N=250, S=100 | 0.038 | 0.039 | | 0.045 | | | 0.023 | | 0.024 |
| N=100, S=40 | 0.055 | 0.049 | | 0.047 | | | 0.029 | | 0.033 |
| N=50, S=20 | 0.051 | 0.044 | | 0.058 | | | 0.021 | | 0.034 |
| N=1000, S=20 | 0.053 | 0.033 | | 0.067 | | | 0.027 | | 0.039 |
| $\boldsymbol{p}_{\boldsymbol{p}\boldsymbol{1}}$**=0.5,** $\boldsymbol{p}_{\boldsymbol{s}\boldsymbol{1}}$**=0.9 (**$\boldsymbol{F}_{\boldsymbol{1}\boldsymbol{,}\boldsymbol{1}}$**=0.643)** | | | | | | | | | |
|  | **psF1** | **psF1-CI** | | **Permutation** | | | **G&G*** | | **Takahashi** |
| $\boldsymbol{p}_{\boldsymbol{p}\boldsymbol{2}}$**=**$\boldsymbol{p}_{\boldsymbol{p}\boldsymbol{1}}$**,** $\boldsymbol{p}_{\boldsymbol{s}\boldsymbol{2}}$**=**$\boldsymbol{p}_{\boldsymbol{s}\boldsymbol{1}}$ |  |  | |  | | |  | |  |
| N=250, S=100 | 0.041 | 0.040 | | 0.039 | | | 0.000 | | 0.000 |
| N=100, S=40 | 0.035 | 0.027 | | 0.050 | | | 0.001 | | 0.001 |
| N=50, S=20 | 0.038 | 0.023 | | 0.042 | | | 0.000 | | 0.002 |
| N=1000, S=20 | 0.033 | 0.022 | | 0.047 | | | 0.006 | | 0.011 |
| $\boldsymbol{p}_{\boldsymbol{p}\boldsymbol{2}}$=$\boldsymbol{p}_{\boldsymbol{s}\boldsymbol{1}}$, $\boldsymbol{p}_{\boldsymbol{s}\boldsymbol{2}}$=$\boldsymbol{p}_{\boldsymbol{p}\boldsymbol{1}}$ |  |  | |  | | |  | |  |
| N=250, S=100 | 0.047 | 0.042 | | 0.032 | | | 0.023 | | 0.024 |
| N=100, S=40 | 0.053 | 0.046 | | 0.036 | | | 0.021 | | 0.026 |
| N=50, S=20 | 0.049 | 0.035 | | 0.041 | | | 0.017 | | 0.030 |
| N=1000, S=20 | 0.055 | 0.040 | | 0.060 | | | 0.027 | | 0.042 |
| $\boldsymbol{p}_{\boldsymbol{p}\boldsymbol{1}}$**=0.9,** $\boldsymbol{p}_{\boldsymbol{s}\boldsymbol{1}}$**=0.7 (**$\boldsymbol{F}_{\boldsymbol{1}\boldsymbol{,}\boldsymbol{1}}$**=0.788)** | | | | | | | | | |
|  | **psF1** | | **psF1-CI** | | **Permutation** | **G&G*** | | **Takahashi** | |
| $\boldsymbol{p}_{\boldsymbol{p}\boldsymbol{2}}$**=**$\boldsymbol{p}_{\boldsymbol{p}\boldsymbol{1}}$**,** $\boldsymbol{p}_{\boldsymbol{s}\boldsymbol{2}}$**=**$\boldsymbol{p}_{\boldsymbol{s}\boldsymbol{1}}$ |  | |  | |  |  | |  | |
| N=250, S=100 | 0.050 | | 0.046 | | 0.052 | 0.045 | | 0.046 | |
| N=100, S=40 | 0.040 | | 0.036 | | 0.057 | 0.035 | | 0.041 | |
| N=50, S=20 | 0.038 | | 0.029 | | 0.042 | 0.028 | | 0.037 | |
| N=1000, S=20 | 0.037 | | 0.028 | | 0.046 | 0.029 | | 0.037 | |
| $\boldsymbol{p}_{\boldsymbol{p}\boldsymbol{2}}$=$\boldsymbol{p}_{\boldsymbol{s}\boldsymbol{1}}$, $\boldsymbol{p}_{\boldsymbol{s}\boldsymbol{2}}$=$\boldsymbol{p}_{\boldsymbol{p}\boldsymbol{1}}$ |  | |  | |  |  | |  | |
| N=250, S=100 | 0.052 | | 0.051 | | 0.044 | 0.038 | | 0.036 | |
| N=100, S=40 | 0.041 | | 0.036 | | 0.034 | 0.020 | | 0.027 | |
| N=50, S=20 | 0.044 | | 0.031 | | 0.054 | 0.015 | | 0.031 | |
| N=1000, S=20 | 0.048 | | 0.026 | | 0.051 | 0.018 | | 0.035 | |

*Power: the probability that CrI does not include zero.

**Table S8.** Power comparison between methods for the difference between two classifiers’ $F_{1}$ scores at the significance level of 0.05 in scenarios with very small $S/N$ ratios.

| $\boldsymbol{p}_{\boldsymbol{p}\boldsymbol{1}}$=0.5, $\boldsymbol{p}_{\boldsymbol{s}\boldsymbol{1}}$=0.8 ($\boldsymbol{F}_{\boldsymbol{1}\boldsymbol{,}\boldsymbol{1}}$=0.615); $\boldsymbol{p}_{\boldsymbol{p}\boldsymbol{2}}$=0.6, $\boldsymbol{p}_{\boldsymbol{s}\boldsymbol{2}}$=0.9 ($\boldsymbol{F}_{\boldsymbol{1}\boldsymbol{,}\boldsymbol{2}}$=0.720) | | | | | | | | | |
| --- | --- | --- | --- | --- | --- | --- | --- | --- | --- |
|  | **psF1** | **psF1-CI** | | **Permutation** | | | **G&G*** | | **Takahashi** |
| N=8000, S=80  S/N=1% | 0.649 | 0.642 | | 0.633 | | | 0.488 | | 0.505 |
| N=8000, S=60  S/N=0.75% | 0.519 | 0.512 | | 0.498 | | | 0.370 | | 0.388 |
| N=8000, S=40  S/N=0.5% | 0.390 | 0.376 | | 0.354 | | | 0.239 | | 0.267 |
| N=8000, S=20  S/N=0.25% | 0.202 | 0.178 | | 0.191 | | | 0.111 | | 0.138 |
| $\boldsymbol{p}_{\boldsymbol{p}\boldsymbol{1}}$**=0.5,** $\boldsymbol{p}_{\boldsymbol{s}\boldsymbol{1}}$**=0.9 (**$\boldsymbol{F}_{\boldsymbol{1}\boldsymbol{,}\boldsymbol{1}}$**=0.643);** $\boldsymbol{p}_{\boldsymbol{p}\boldsymbol{2}}$**=0.6,** $\boldsymbol{p}_{\boldsymbol{s}\boldsymbol{2}}$**=0.9 (**$\boldsymbol{F}_{\boldsymbol{1}\boldsymbol{,}\boldsymbol{2}}$**=0.720)** | | | | | | | | | |
|  | **psF1** | | **psF1-CI** | | **Permutation** | **G&G*** | | **Takahashi** | |
| N=8000, S=80  S/N=1% | 0.448 | | 0.439 | | 0.436 | 0.282 | | 0.294 | |
| N=8000, S=60  S/N=0.75% | 0.368 | | 0.358 | | 0.347 | 0.214 | | 0.230 | |
| N=8000, S=40  S/N=0.5% | 0.242 | | 0.232 | | 0.230 | 0.121 | | 0.150 | |
| N=8000, S=20  S/N=0.25% | 0.127 | | 0.106 | | 0.132 | 0.041 | | 0.069 | |
| $\boldsymbol{p}_{\boldsymbol{p}\boldsymbol{1}}$**=0.9,** $\boldsymbol{p}_{\boldsymbol{s}\boldsymbol{1}}$**=0.7 (**$\boldsymbol{F}_{\boldsymbol{1}\boldsymbol{,}\boldsymbol{1}}$**=0.788);** $\boldsymbol{p}_{\boldsymbol{p}\boldsymbol{2}}$**=0.9,** $\boldsymbol{p}_{\boldsymbol{s}\boldsymbol{2}}$**=0.9 (**$\boldsymbol{F}_{\boldsymbol{1}\boldsymbol{,}\boldsymbol{2}}$**=0.9)** | | | | | | | | | |
|  | **psF1** | | **psF1-CI** | | **Permutation** | **G&G*** | | **Takahashi** | |
| N=8000, S=80  S/N=1% | 0.720 | | 0.699 | | 0.719 | 0.696 | | 0.701 | |
| N=8000, S=60  S/N=0.75% | 0.602 | | 0.579 | | 0.594 | 0.572 | | 0.584 | |
| N=8000, S=40  S/N=0.5% | 0.434 | | 0.396 | | 0.403 | 0.393 | | 0.410 | |
| N=8000, S=20  S/N=0.25% | 0.222 | | 0.190 | | 0.201 | 0.174 | | 0.216 | |

*Power: the probability that CrI does not include zero.

**Table S9.** Confusion matrices for standard screening and cfDNA testing in the diagnosis of trisomy 21.

|  |  | STD Screening | |  | cfDNA Testing | |  |
| --- | --- | --- | --- | --- | --- | --- | --- |
|  |  | Test (-) | Test (+) |  | Test (-) | Test (+) |  |
| **Actual** | No trisomy 21  (D-) | 14949 | 854 |  | 15794 | 9 | 15803 |
|  | Trisomy 21 (D+) | 8 | 30 |  | 0 | 38 | 38 |
|  |  | 14957 | 884 |  | 15794 | 47 | 15841 |

**Table S10.** Confusion matrices for three models in the prediction of postoperative urinary retention.

|  |  | Model 1 | |  | Model 2 | |  | Model 3 | |  |
| --- | --- | --- | --- | --- | --- | --- | --- | --- | --- | --- |
|  |  | Test (-) | Test (+) |  | Test (-) | Test (+) |  | Test (-) | Test (+) |  |
| **Actual** | No POUR  (D-) | 150 | 26 |  | 68 | 108 |  | 120 | 56 | 176 |
|  | POUR  (D+) | 30 | 29 |  | 9 | 50 |  | 16 | 43 | 59 |
|  |  | 180 | 55 |  | 77 | 158 |  | 136 | 99 | 235 |

**References**

1. Porche K, Maciel CB, Lucke-Wold B, et al. Preoperative prediction of postoperative urinary retention in lumbar surgery: a comparison of regression to multilayer neural network. *J Neurosurg Spine*. 2022;36:32-41.
